# The essential roles of FXR in diet and age influenced metabolic changes and liver disease development: a multi-omics study

**DOI:** 10.1186/s40364-023-00458-9

**Published:** 2023-02-18

**Authors:** Guiyan Yang, Prasant K. Jena, Ying Hu, Lili Sheng, Shin-Yu Chen, Carolyn M. Slupsky, Ryan Davis, Clifford G. Tepper, Yu-Jui Yvonne Wan

**Affiliations:** 1 Department of Pathology and Laboratory Medicine, University of California, Davis Health. Room 3400B, Research Building III, 4645 2nd Ave, Sacramento, CA 95817 USA; 2 Department of Nutrition, University of California, Davis, CA USA; 3 Department of Biochemistry and Molecular Medicine, University of California, Davis, Sacramento, CA USA

**Keywords:** Liver, Metabolic disease, Nonalcoholic fatty liver disease, Nonalcoholic steatohepatitis, Hepatocellular carcinoma, Bile acid, Bile acid receptor, Gut microbiota

## Abstract

**Background:**

Aging and diet are risks for metabolic diseases. Bile acid receptor farnesoid X receptor (FXR) knockout (KO) mice develop metabolic liver diseases that progress into cancer as they age, which is accelerated by Western diet (WD) intake. The current study uncovers the molecular signatures for diet and age-linked metabolic liver disease development in an FXR-dependent manner.

**Methods:**

Wild-type (WT) and FXR KO male mice, either on a healthy control diet (CD) or a WD, were euthanized at the ages of 5, 10, or 15 months. Hepatic transcriptomics, liver, serum, and urine metabolomics as well as microbiota were profiled.

**Results:**

WD intake facilitated hepatic aging in WT mice. In an FXR-dependent manner, increased inflammation and reduced oxidative phosphorylation were the primary pathways affected by WD and aging. FXR has a role in modulating inflammation and B cell-mediated humoral immunity which was enhanced by aging. Moreover, FXR dictated neuron differentiation, muscle contraction, and cytoskeleton organization in addition to metabolism. There were 654 transcripts commonly altered by diets, ages, and FXR KO, and 76 of them were differentially expressed in human hepatocellular carcinoma (HCC) and healthy livers. Urine metabolites differentiated dietary effects in both genotypes, and serum metabolites clearly separated ages irrespective of diets. Aging and FXR KO commonly affected amino acid metabolism and TCA cycle. Moreover, FXR is essential for colonization of age-related gut microbes. Integrated analyses uncovered metabolites and bacteria linked with hepatic transcripts affected by WD intake, aging, and FXR KO as well as related to HCC patient survival.

**Conclusion:**

FXR is a target to prevent diet or age-associated metabolic disease. The uncovered metabolites and microbes can be diagnostic markers for metabolic disease.

**Supplementary Information:**

The online version contains supplementary material available at 10.1186/s40364-023-00458-9.

## Background

Diet and aging are major risk factors for metabolic diseases including non-alcoholic steatohepatitis (NASH), which leads to the development of hepatocellular carcinoma (HCC) [1, 2]. Currently, there is no drug that can be used to treat NASH, and the outcome of HCC treatment remains unsatisfactory. Early detection and identification of treatment targets are essential to moving the field forward.

With the use of fecal transplantation, antibiotic treatment, probiotic and prebiotic interventions, as well as dietary supplementation, it is now clear that gut microbes and their metabolites contribute to metabolic disease development [3–6]. One of the established mechanisms includes that diet-associated dysbiosis accompanied by dysregulated bile acid (BA) synthesis leading to IL17A production and systemic inflammation [7–9]. Thus, diet- and aging-associated gut microbes and metabolites can be biomarkers for metabolic liver disease development.

BAs are synthesized from hepatic metabolism of dietary cholesterol and are further metabolized by bacterial enzymes to generate secondary BAs in the intestine [10]. The beneficial or toxic effects of BAs depend on their composition and concentration. The metabolic, detoxification, and immune functions of BAs are mediated through their receptors, among which FXR (farnesoid X receptor) and GPBAR1 (G protein-coupled bile acid receptor 1) have been studied extensively [10]. Because FXR regulates BA homeostasis, its knockout (KO) in mice causes BA synthesis dysregulation and leads to the development of non-alcoholic fatty liver (5 months old), which progresses into NASH (10 months old) and HCC (15 months old) even when FXR KO mice consume a healthy diet [11, 12].

Patients who have liver cirrhosis or HCC have reduced FXR [13]. Our previous study has reported that Western diet (WD) intake facilitates the tumorigenesis of FXR KO mice in a sex-dependent manner [5, 14]. Moreover, WD-induced systemic inflammation is accompanied by BA receptor deactivation [7, 14]. In contrast, probiotics-prevented hepatic inflammation is associated with the restoration of BA receptor-regulated signaling [6]. These findings revealed the significance of dysregulated BA, which is noted in FXR KO mice, in contributing to metabolic liver disease development.

The current study used multi-omics approaches to uncover the impact of diet, age, and FXR functional status on gut microbiota, metabolites (liver, urine, serum), and hepatic phenotypes based on transcriptomic pathways. Our novel data revealed the essential roles of FXR in combating diet and age-associated metabolic liver disease development. Additionally, the data revealed the molecular signatures within the gut-liver axis implicated in the development of non-alcoholic fatty liver disease (NAFLD) and HCC.

## Methods

### Specimens and animals

The specimens used were derived from mice with established phenotypes [4, 5, 14, 15]. Wild-type (WT) and FXR KO [16] male mice were specific pathogen-free with the genetic background of C57BL/6 N. Mice were fed either a healthy control diet (CD, TD.140415; 5.2% fat, 12% sucrose, and 0.01% cholesterol, w/w) or a Western diet (WD, TD.140414; 21.2% fat, 34% sucrose, and 0.2% cholesterol, w/w) (Harlan Teklad, Madison, WI) since weaning (3 weeks) and euthanized at the age of 5, 10, and 15 months. Diet and water were provided ad libitum. Experiments were conducted in accordance with the NIH Guide for the Care and Use of Laboratory Animals under protocols approved by the Institutional Animal Care and Use Committee of the University of California, Davis (Sacramento, CA).

### Untargeted metabolomic study

Hepatic metabolites (*n* = 6/group) were analyzed by gas chromatography–time-of-flight mass spectrometry (GC-TOF-MS) at the West Coast Metabolomics Center (University of California, Davis). All database entries in BinBase were filtered and matched against a Mass Spectral Library of 1200 authentic metabolites spectra with retention index and mass spectrum information or against the National Institution of Standards and Technology library.

### Quantification of serum and urine metabolites

Serum and urine metabolites (*n* = 6/group) were quantified by NMR using published methods (9). Briefly, samples were filtered by Amicon Ultra-0.5 mL centrifugal filter. Avance 600 MHz NMR spectrometer (Bruker, Billerica, MA) equipped with a SampleJet was used for NMR data acquisition. The concentrations of serum and urine metabolites were normalized by log transformation [15].

### Quantification of hepatic bile acids

Hepatic bile acids (*n* = 6/group) were quantified using a Prominence™ UFLC system (Shimadzu, Kyoto, Japan) coupled to an API 4000 QTRAP mass spectrometer (Sciex, Redwood City, CA) operated in the negative ionization mode, as previously described [14].

### RNA sequencing and data processing

Hepatic RNA was extracted (*n* = 4) using TRIzol reagent (Invitrogen, Carlsbad, CA). The liver specimens used were from the same cohort of mice used in previous publications [4, 5, 14, 15]. RNA sequencing was performed by Novogene Co., LTD (Sacramento, CA). Raw sequence reads (FASTQ) data were mapped to the reference mouse transcriptome index (GRCm39/mm39, GENCODE release M27) and quantified with Salmon (version 1.4.0) pseudoaligner [17]. Gene-level counts were imported with *tximport* [18] and differential expression analysis was performed with DESeq2 (version 1.18) using the lfcShrink function [19] in R 4.03.

### Human databases

RNA sequencing data (normalized RSEM values) for 371 liver hepatocellular carcinoma and 50 normal control samples were sourced from The Cancer Genome Atlas (TCGA) database.

### Bioinformatics

Principal component analysis (PCA), statistical analysis, and quantitative enrichment analysis for metabolites were conducted with MetaboAnalyst 5.0 (https://www.metaboanalyst.ca). PCA of transcriptomic data was performed using R 4.2.0. Normalized read counts were used for Gene Set Enrichment Analysis (GSEA) [20]. Pathway analyses of the differentially expressed genes (DEGs) were performed using Metascape (http://metascape.org). The overall survival rate was assessed using OncoLnc (http://www.oncolnc.org/).

### Statistics

Metabolite levels were statistically significant if the uncorrected *p* value < 0.05 and the false discovery rate (FDR) corrected *p* value < 0.1. Genes with FDR corrected *p*-value < 0.05 and absolute fold change ≥2 were considered as differentially expressed. Spearman’s correlation was used to assess the relationship between hepatic features (transcripts and metabolites) and non-hepatic features (serum and urine metabolites as well as gut microbiota at the genus level), and a significant correlation was defined when the Hochberg-adjusted *p* value < 0.05. Data are expressed as the mean ± SD. *p < 0.05 was considered significantly different.

## Results

### Hepatic phenotypes

Hepatic phenotypes have been reported [3–5, 14], and the findings are summarized as follows. WD consumption induced steatosis in WT and FXR KO mice of all ages in a time-dependent manner. Healthy CD-fed 5-month-old FXR KO mice developed steatosis which increased in severity with age [14]. WD intake exacerbated the pathology in FXR KO mice, resulting in the development of NASH at 10 months of age [5], and liver tumors at 15 months of age [4]. In comparison with healthy CD-fed WT mice, serum cholesterol and triglyceride concentrations were substantially increased in WD-fed FXR KO mice as early as 5 months of age (Fig. S1).

### WD-altered hepatic transcripts and pathways were largely dependent on FXR function

**Fig. 1:**
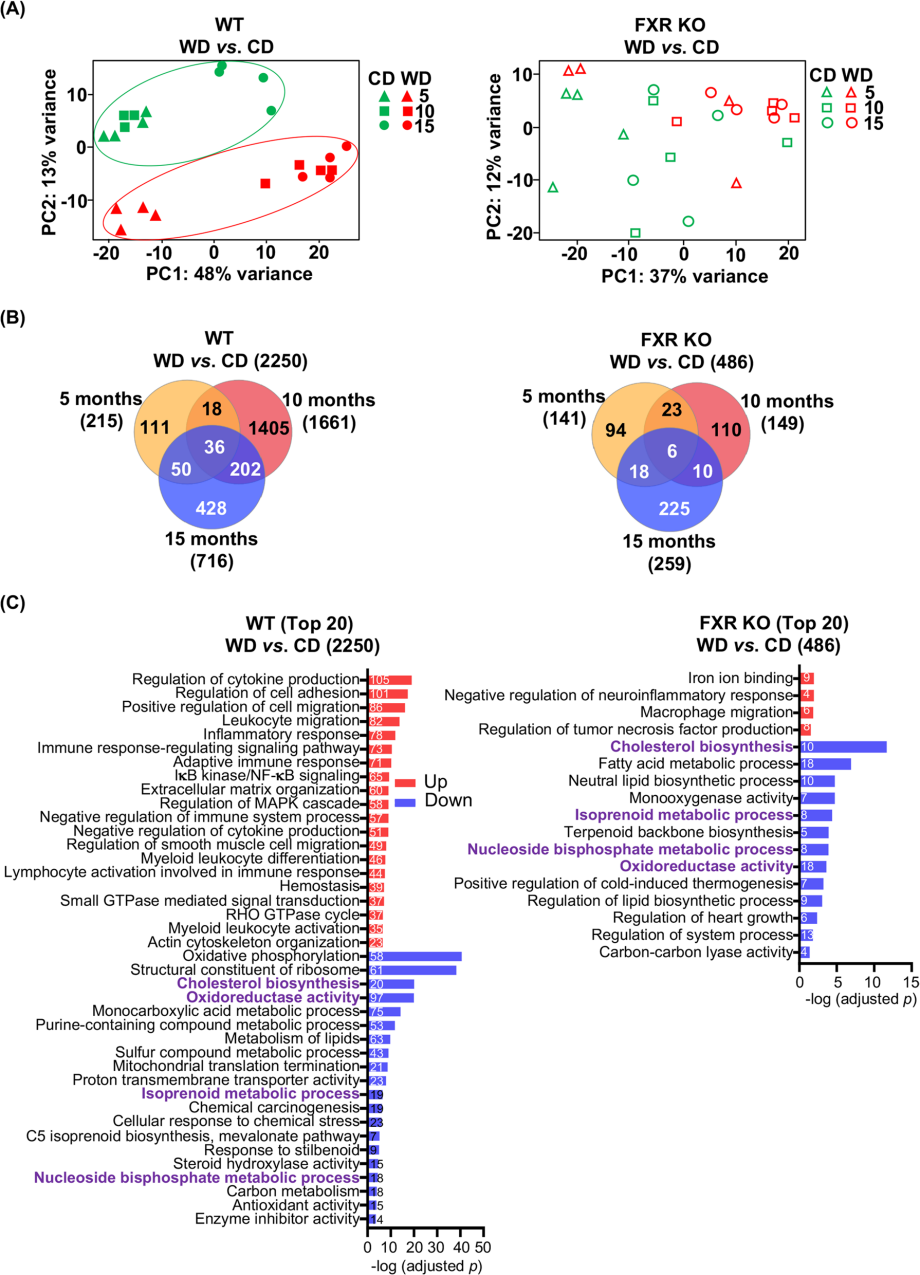
The effects of diets on hepatic transcriptomes of WT and FXR KO mice with different ages. **A** Principal component analyses of liver transcriptomes of 5-, 10-, and 15-month-old WT and FXR KO mice fed with either a CD or WD since weaning. **B** Venn diagrams show the numbers of genes differentially expressed due to diet (WD *vs*. CD) intake in WT or FXR KO mice. **C** Pathway enrichment analyses for up and downregulated genes affected by differential diet intake using Metascape. The top 20 pathways are shown (adjusted *p* value < 0.05). WD-regulated pathways shared by WT and FXR KO mice are in purple. The numbers of differentially expressed genes in each pathway are shown in the bars

Principal component analysis (PCA) of hepatic transcriptomes showed that CD- and WD-fed mice formed two clusters in WT mice but not in FXR KO mice (Fig. 1A). In addition, the number of differentially expressed genes (DEGs) based on diets was much smaller in FXR KO mice (486) than in WT mice (2250) (Fig. 1B). There were 36 transcripts consistently altered by WD in WT mice irrespective of their ages, but only 6 (*Scd3*, *Cidec*, *Csad*, *Cyp39a1*, *Dntt*, and *9130409I23Rik*) were found in FXR KO mice (Fig. S2). Those 36 transcripts might represent the hallmarks of WD intake. The functions of those transcripts are summarized in Table S1.

Pathway analyses were performed for the 2250 DEGs from WT and 486 DEGs in FXR KO mice. WD increased cytokine production and inflammation but downregulated oxidative phosphorylation (OXPHOS) only in WT mice. Common pathways that changed due to WD intake in both genotypes were: downregulation of genes implicated in cholesterol biosynthesis, oxidoreductase activity, isoprenoid metabolic processes, and nucleoside bisphosphate metabolic processes (Fig. 1C, *p* < 0.05 and enrichment factor ≥ 1.5). In FXR KO mice, WD uniquely upregulated iron binding but downregulated fatty acid metabolism genes (Fig. 1C). Together, when FXR is deactivated, many WD-regulated genes and pathways were no longer differentially found, indicating the similarity between WD intake and FXR inactivity. Diet-changed transcripts in WT but not in FXR KO mice may be direct FXR targets or due to secondary effects of FXR deactivation. Additionally, base don *Nr1h4* FXR cistrome [21], and found that *Cebpa*, *Cebpb*, *Clock*, *Per1*, *Rarb*, and *Rora* are FXR direct target genes.

### Dietary effects on the liver, serum, and urine metabolome as well as gut microbiota in WT and FXR KO mice

**Fig. 2:**
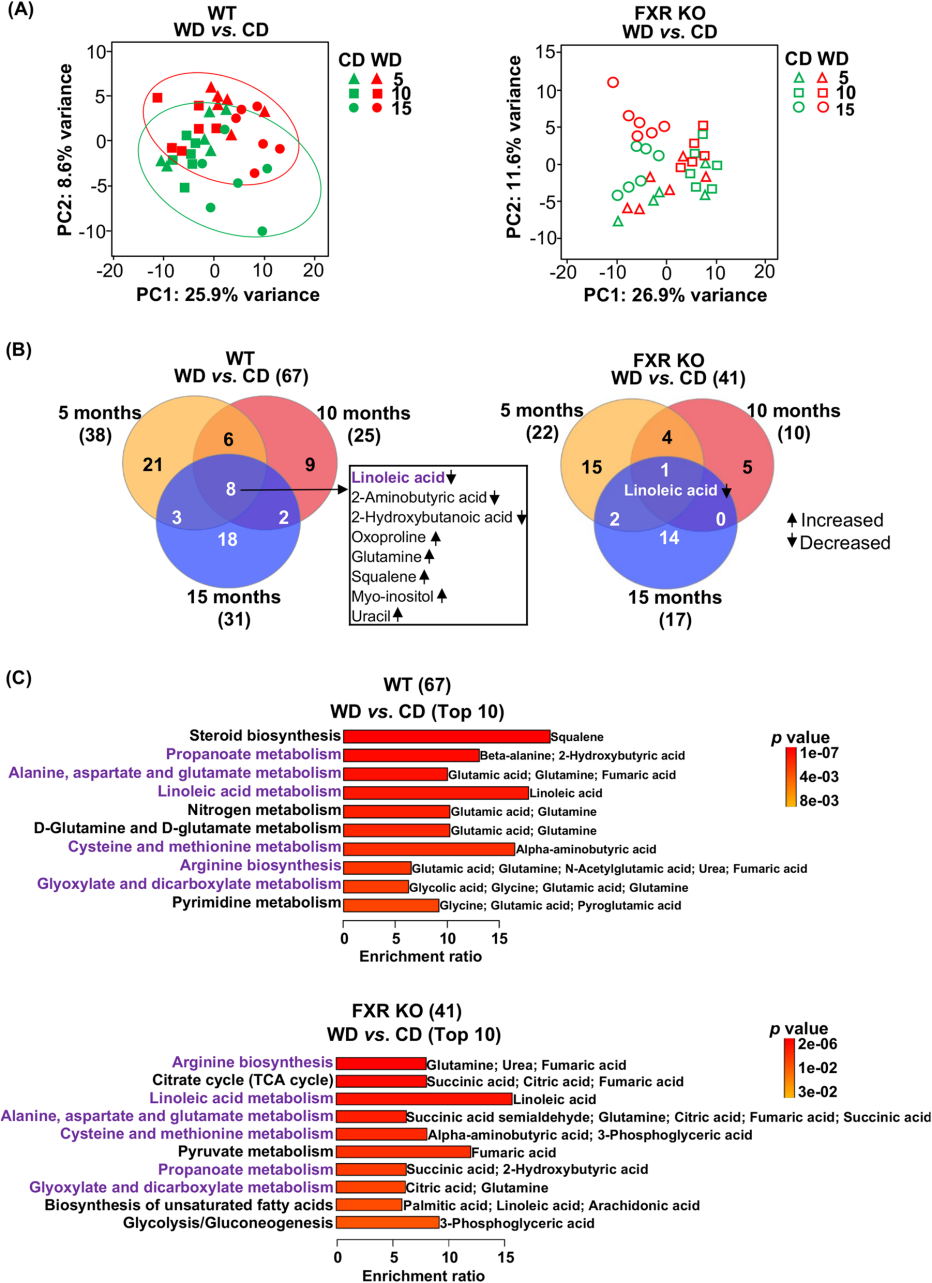
The effects of diets on hepatic metabolomes in WT and FXR KO mice. **A** Principal component analyses of liver metabolomes in WT and FXR KO mice **B** Venn diagrams show the numbers of distinct and overlapping metabolites that were changed by differential diet intake in 3 age groups (raw *p* value < 0.05 and FDR < 0.1). **C** Quantitative enrichment analyses of diet-altered metabolites. The top 10 pathways are shown (*p* value < 0.05). Common metabolic changes in metabolites and pathways found in both genotypes are marked in purple

Similar to the transcriptome results, PCA of the hepatic metabolome datasets revealed that the effects of diet were more distinctive in WT than in FXR KO mice (Fig. 2A). The number of metabolite changes due to differential dietary intake was larger in WT (67) than that in FXR KO (41) mice (Fig. 2B). The diet-altered metabolites commonly or specifically found in WT and FXR KO mice are shown in Fig. S3. Irrespective of ages, WD intake increased hepatic myo-inositol, squalene, glutamine, oxoproline, and uracil but reduced 2-hydroxybutanoic acid, 2-aminobutyric acid, and linoleic acid in WT mice. In FXR KO mice, only hepatic linoleic acid was reduced by WD intake in those 3 age groups (Fig. 2B). Quantitative enrichment analysis showed that arginine biosynthesis, propanoate metabolism, alanine, aspartate and glutamate metabolism, linoleic acid metabolism, cysteine and methionine metabolism, and glyoxylate and dicarboxylate metabolism were altered by WD for both genotypes (purple, Fig. 2C). For FXR KO mice, WD intake altered citrate cycle (succinic acid, citric acid, fumaric acid), pyruvate metabolism (fumaric acid), biosynthesis of unsaturated fatty acids (palmitic acid, linoleic acid, arachidonic acid), and glycolysis/gluconeogenesis (3-phosphoglyceric acid) (black, Fig. 2C).

By contrast, the impact of FXR KO on hepatic bile acids and serum metabolites due to differential diet intake was not obvious when all ages were considered together based on PCA (Fig. S4, S5). However, the impact of diet on urine metabolites was obvious irrespective of age in both genotypes suggesting the specificity of urine metabolites to detect dietary differences (Fig. S6).

Interestingly, upon PCA of the cecal microbiota, the dietary differences were apparent for younger mice (5 and 10 months old, triangles and squares), but not in 15-month-old (circles) WT mice, indicating the dietary influence on the gut microbiota was affected by feeding duration or aging (Fig. S7). In contrast, in FXR KO mice, CD and WD groups formed two distinct clusters suggesting the usefulness of microbiota to differentiate FXR status (Fig. S7).

### The significance of FXR on hepatic transcriptomic changes based on ages

The PCA plot shown in Fig. 1A suggests that WD intake may facilitate liver aging. Specifically, CD-fed 5- and 10-month-old mice clustered distinctly from the 15-month-old mice. However, in WD-fed mice, the 10- and 15-month-old groups formed a cluster. Thus, age-related changes in the hepatic transcriptome were accelerated in WD-fed mice.

**Fig. 3:**
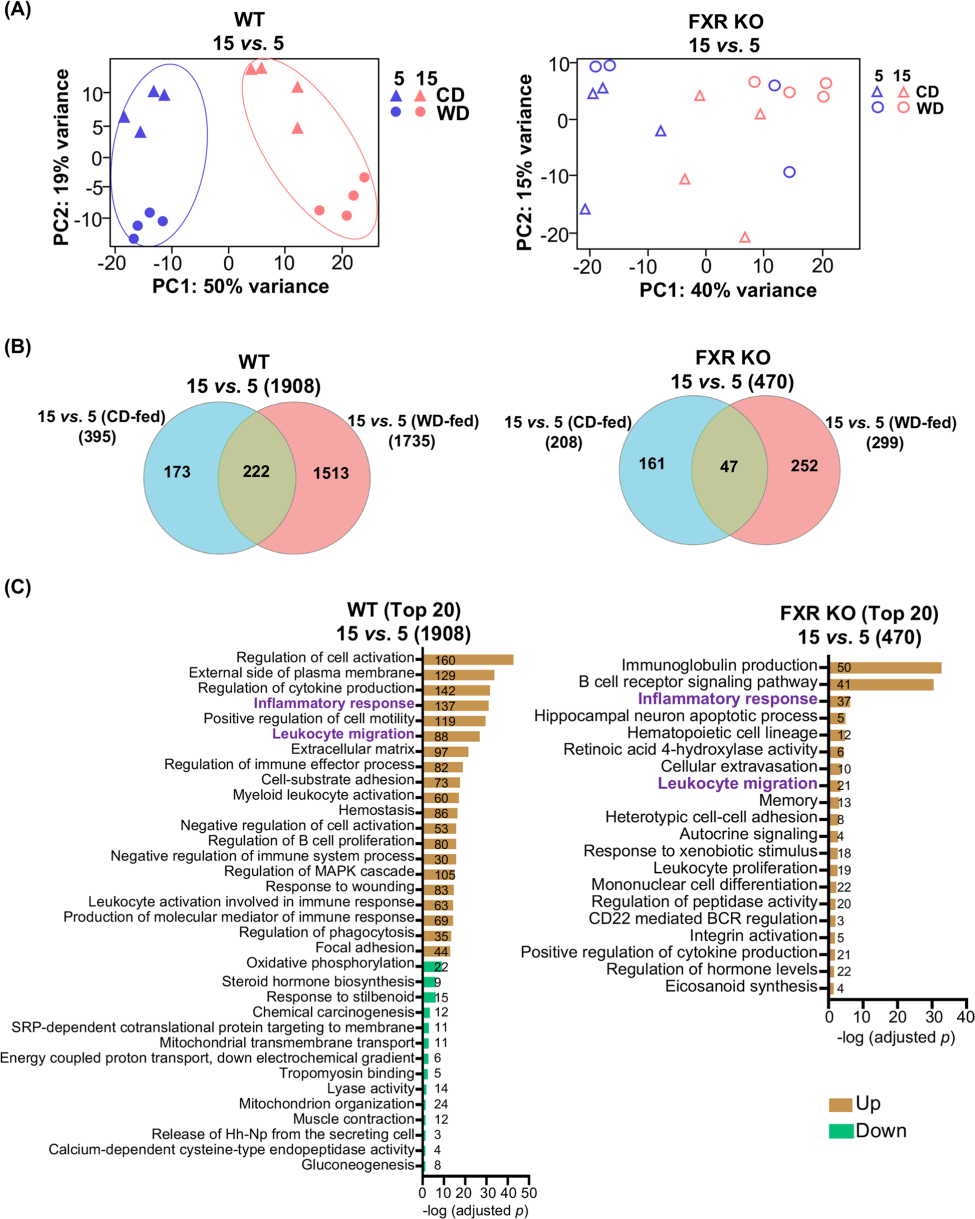
The effects of ages on hepatic transcriptomes in WT and FXR KO mice on different diets. **A** Principal component analyses of liver transcriptomes in WT and FXR KO mice fed with either a CD or WD. **B** Venn diagrams show the numbers of genes differentially expressed due to age (15 *vs*. 5) in WT or FXR KO mice (fold change ≥2 and adjusted *p* value < 0.05). **C** Pathway enrichment analyses for up and downregulated genes affected by age based on Metascape. The top 20 pathways are shown (adjusted *p* value < 0.05). Age-regulated pathways shared by WT and FXR KO mice are in purple. The numbers of differentially expressed genes in each pathway are shown in the bars

The effects of age (15 *vs*. 5 months old) on the transcriptomes were further analyzed. PCA of hepatic transcriptomes differentiated ages in WT but not in FXR KO mice (Fig. 3A). Many more DEGs were found in WT (1908), due to age differences, than in FXR KO (470) mice (Fig. 3B). Some of those genes are FXR direct target genes and the examples are Onecult1 and Rarb [21]. Similar to the dietary effects, more genes involved in the inflammatory response and leukocyte migration functional categories upregulated by aging were in WT than in FXR KO mice (Fig. 3C). In FXR KO mice, aging uniquely upregulated the hematopoietic cell lineage and B cell receptor signaling pathways (Fig. 3C). In addition, age-downregulated OXPHOS genes were only noted in WT mice, highlighting the significance of FXR in OXPHOS.

### Age-related changes of metabolites and cecal microbiota in WT and FXR KO mice

**Fig. 4:**
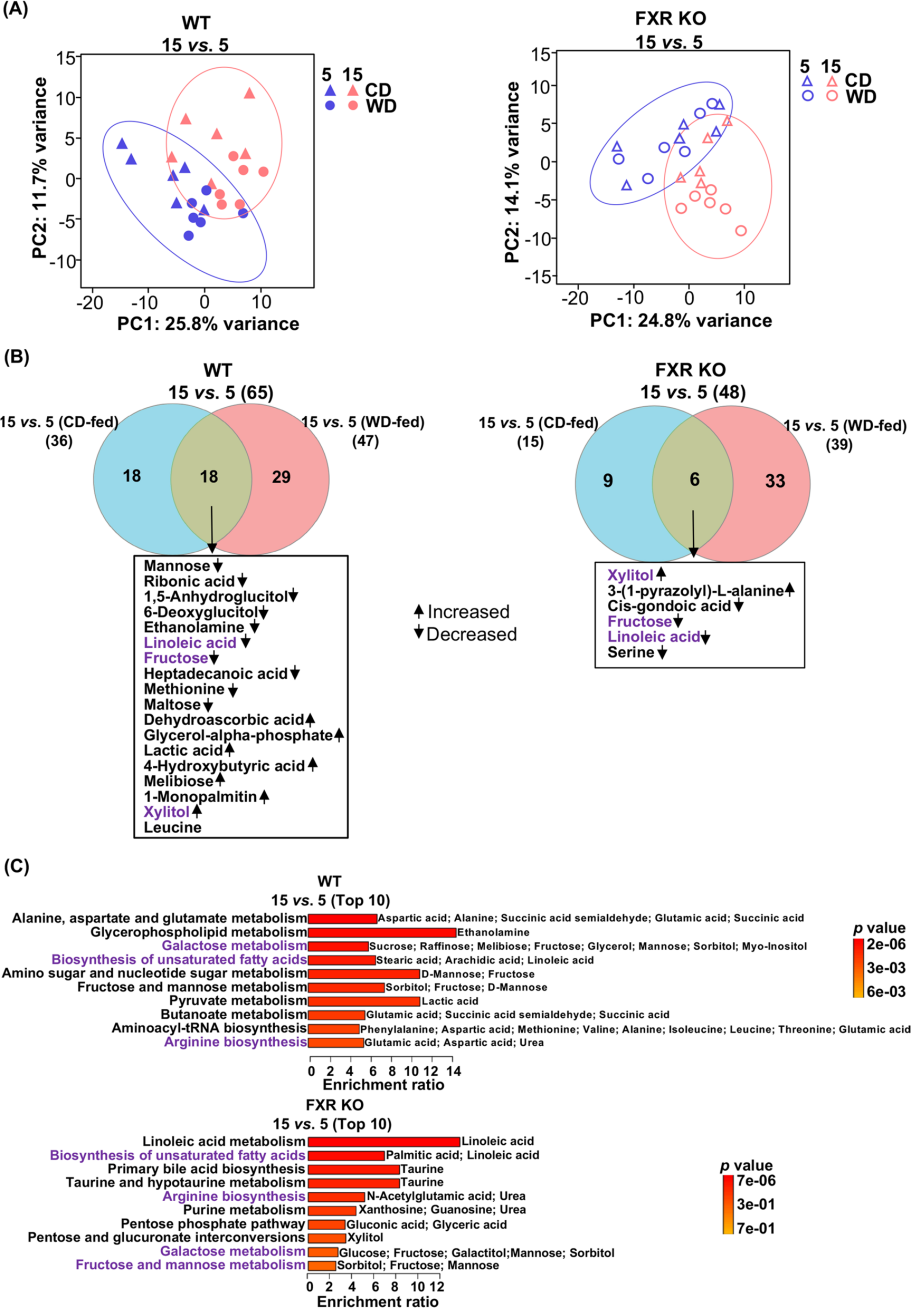
The effects of ages on hepatic metabolomes in WT and FXR KO mice. **A** Principal component analyses of liver metabolomes of WT and FXR KO mice. **B** Venn diagrams show the numbers of distinct and overlapping metabolites that were changed by age (raw *p* value < 0.05 and FDR < 0.1). **C** Quantitative enrichment analyses of age altered metabolites. The top 10 pathways are shown (*p* value < 0.05). Common metabolic changes in metabolites and pathways found in both WT and FXR KO mice are marked in purple

Although the number of liver metabolites altered by age in FXR KO (*n* = 48) was smaller than that in WT mice (*n* = 65), two clusters were still noted based on age (15 *vs*. 5) in both WT and FXR KO mice as shown in Fig. 4A-B. In both genotypes, aging elevated hepatic xylitol but reduced fructose and linoleic acid irrespective of the type of diet (Fig. 4B, S8). The top 10 metabolic pathways affected by age in each genotype are summarized in Fig. 4C. Among them, biosynthesis of unsaturated fatty acids, arginine biosynthesis, galactose metabolism, and fructose and mannose metabolism were affected by age in both genotypes (Fig. 4C). Moreover, aging decreased hepatic ethanolamine and metabolites involved in the aminoacyl-tRNA biosynthesis in WT mice revealing the significance of FXR in regulating hepatic amino acid/peptide synthesis (Fig. 4C, S8).

Hepatic bile acid profile, serum, and urine metabolites were also differentiated by age effects in both genotypes (Fig. S9, S10, S11), but different metabolites altered by age were found between WT and FXR KO mice. Collectively, the data suggest that FXR plays a significant role in age-linked metabolic changes. Consistent with transcriptomic changes, the age-mediated differences in the cecal microbiota at the genus level were not obvious in FXR KO mice (Fig. S12A). Taken together, the findings suggest that FXR dictates the age-associated gut microbiota community structure.

### Molecular signatures alterations due to FXR deficiency

**Fig. 5:**
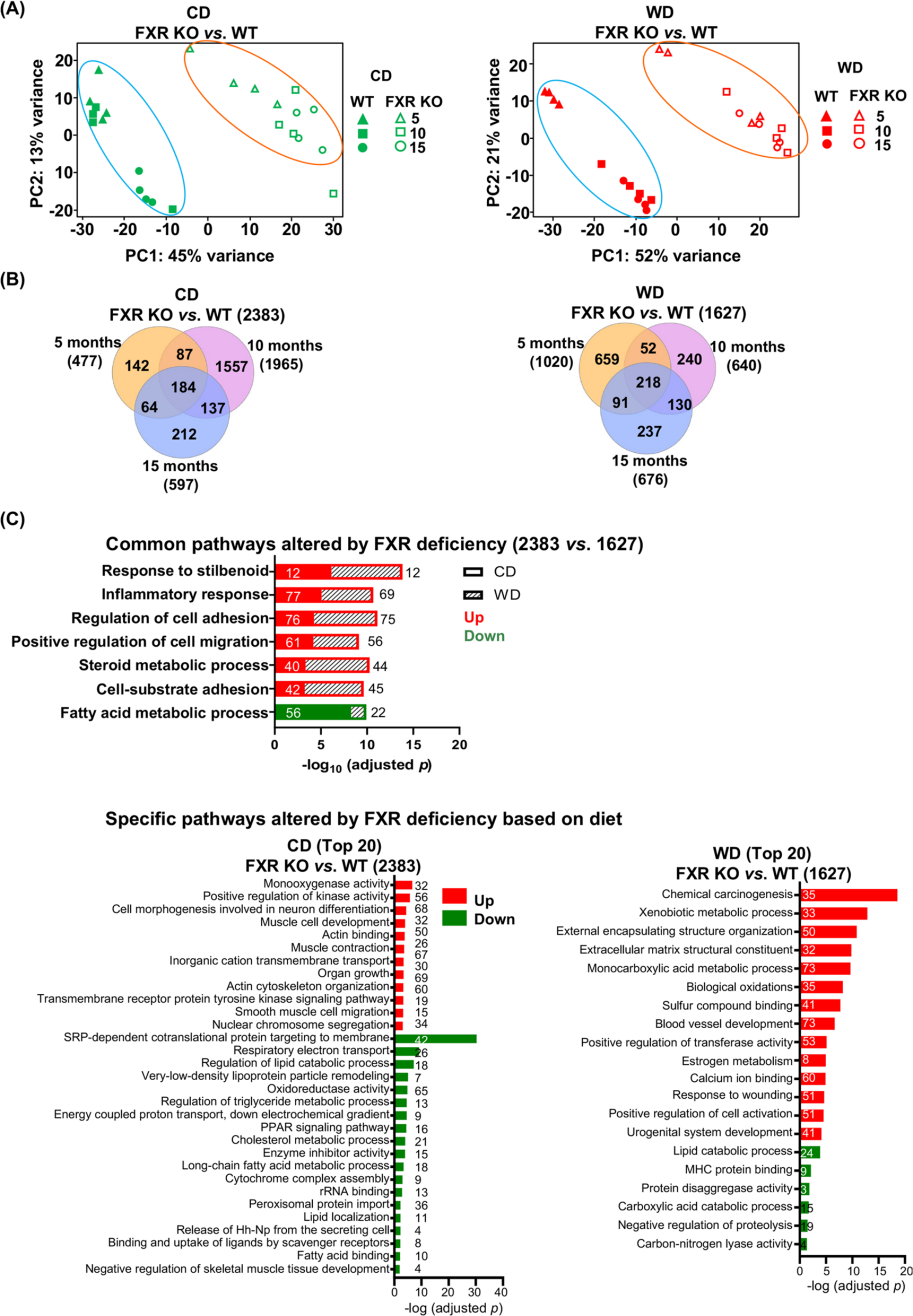
The effects of FXR KO on hepatic transcriptomes. **A** Principal component analyses of liver transcriptomes of WT and FXR KO mice fed with differential diets until 5, 10, and 15 months old. **B** Venn diagrams show the numbers of genes differentially expressed due to FXR KO (fold change ≥2 and adjusted *p* value < 0.05). **C** Pathways and numbers of genes that were up or down regulated by FXR KO found in both CD- and WD-fed mice based on Metascape. The top 20 pathways are shown (adjusted *p* value < 0.05). Common and specific transcriptomic changes (pathways and numbers of differentially expressed genes in each pathway) due to FXR KO in CD- and WD-fed mice

PCA revealed that FXR inactivation shifted the hepatic transcriptomes of both CD- and WD-fed mice of different ages, as revealed by the alteration in expression of 2383 and 1627 transcripts in CD and WD groups, respectively (Fig. 5A). In both dietary groups, common pathways that shifted due to lack of FXR are shown in Fig. 5C. Remarkably, when mice were on a WD, FXR deactivation led to enhanced chemical carcinogenesis, extracellular matrix structural constituent, etc., suggestive of hepatocarcinogenicity impacted by WD in combination with lack of FXR (Fig. 5C).

**Fig. 6:**
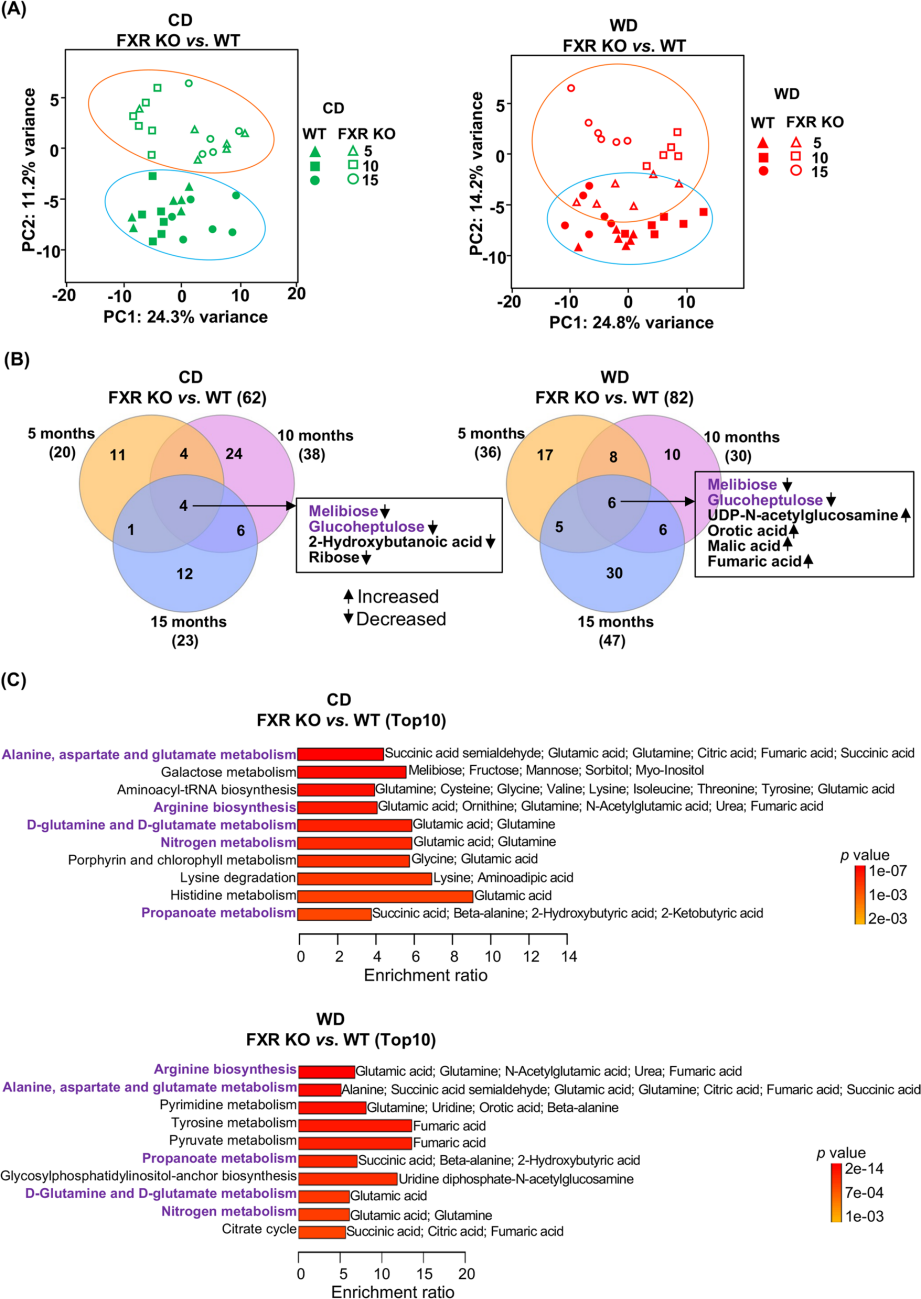
The effects of FXR KO on hepatic metabolomes. **A** Principal component analyses of hepatic metabolomes of WT and FXR KO mice fed with differential diets until 5, 10, and 15 months old. **B** Venn diagrams show the numbers of distinct and overlapping metabolites that were altered by FXR KO in CD- and WD-fed mice (raw *p* value < 0.05 and FDR < 0.1). **C** Quantitative enrichment analyses of FXR KO altered metabolites. The top 10 pathways are shown (*p* value < 0.05). Common pathways in both CD- and WD-fed mice are marked in purple

For hepatic metabolites, FXR KO mice had reduced sugars (e.g., melibiose and glucoheptulose) irrespective of diet or age (Fig. 6B, S13). Additionally, lack of FXR increased hepatic fumaric acid, malic acid, and parabanic acid (Fig. S13A). There was a combined effect of diet, age, and FXR KO in which more changes were noted in WD-fed FXR KO mice at the age of 15 months when the mice had liver tumors [4]. For example, hepatic orotic acid, 3-phosphoglycerate, levoglucosan, alanine-alanine, 3,6-anhydrous-D-galactose, glucose, galactitol, xylitol, erythritol, and xanthosine were accumulated in 15-month-old WD-fed FXR KO mice, which were not seen in WT mice of the same age on the WD (Fig. S13B; highlighted in red). FXR KO altered amino acid and propanoate metabolism (purple, Fig. 6C). Pyrimidine metabolism, tyrosine metabolism, and pyruvate metabolism as well as glycosylphosphatidylinositol-anchor biosynthesis and citrate cycle pathways affected by lacking FXR were uniquely found in WD-fed mice (Fig. 6C; down, marked in black). These changes may reflect the cancer phenotype developed by FXR deactivation and WD intake. In addition, the FXR KO effect on hepatic bile acid profiles in mice fed with either CD or WD is shown in Fig. S14.

Further analysis revealed that changes in FXR KO-linked serum metabolites were only apparent in the CD group revealing the overlapping effects of WD intake and FXR KO (Fig. S15A). Serum 2-hydroxybutyrate increased in FXR KO mice at all ages irrespective of diets (purple, Figs. S15B, S16). In contrast to serum metabolites, PCA plots showed that urine metabolites clustered into two distinct groups based on the mouse genotypes in both CD and WD-fed mice (Fig. S17). FXR KO increased urine 2-hydroxyvalerate and acetoacetate but reduced taurine, creatinine, and trimethylamine (TMA) irrespective of diets (purple, Figs. S17, S18). In addition to urine metabolites, the cecal microbiota distinguished WT and FXR KO (Fig. S19). Notably, cecal *Bacteroides* were increased by FXR deactivation regardless of the provided diet type (purple, Fig. S19B-C).

### Common changes due to WD intake, aging, and FXR KO

**Fig. 7:**
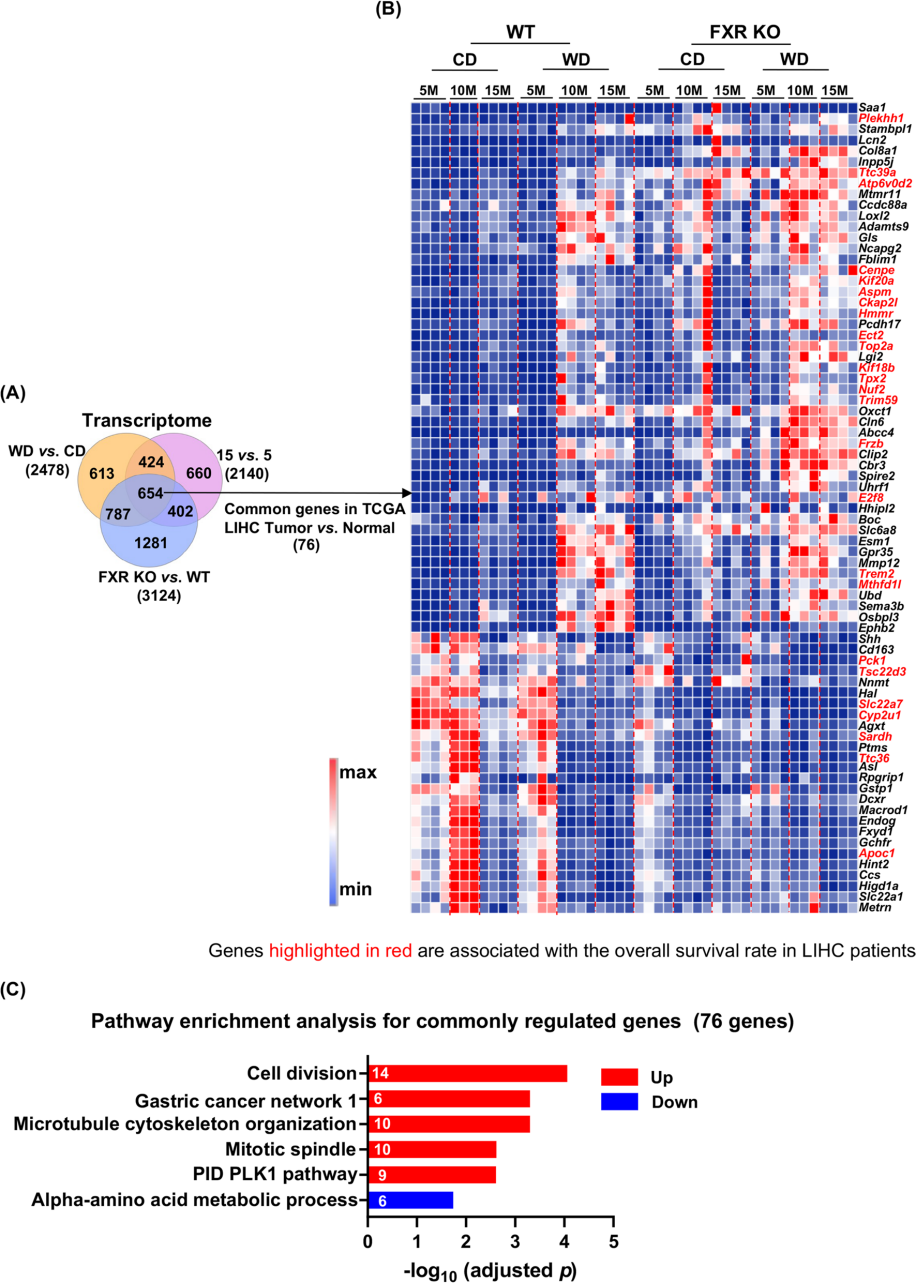
Common changes of hepatic transcripts due to differential diet intake, ages, and FXR KO in association with the expression of genes implicated in human HCC patient survival. **A** Venn diagram shows the numbers of common genes (transcripts) (fold change ≥2 and adjusted *p* value < 0.05). **B** A heatmap reveals transcript levels of mouse genes implicated in hepatic metabolic disease (*n* = 76) and human genes differentially expressed in human HCC and healthy livers. **C** Pathway enrichment analyses of 76 mouse hepatic genes that are implicated in human HCC survival using Metascape (adjusted *p* value < 0.05). The numbers of differentially expressed genes in each pathway are shown in the bar graph. LIHC, liver hepatocellular carcinoma

The expression levels of a total of 654 transcripts were commonly altered due to differential diet intake, ages, and FXR functionality (Fig. 7A). Among them, 76 transcripts have altered expression levels in human HCC specimens compared with healthy livers using the TCGA database (Fig. 7A). Pathway analysis of those 76 transcripts revealed their roles in cell division, mitotic spindle, and cancer development (Fig. 7C). Furthermore, 18 out of those 76 transcripts were upregulated in HCC and their expression levels were positively associated with poor overall survival in HCC patients (Table 1). Those 18 genes are *PLEKHH1*, *TTC39A*, *ATP6V0D2*, *CENPE*, *KIF20A*, *ASPM*, *CKAP2L*, *HMMR*, *ECT2*, *TOP2A*, *KIF18B*, *TPX2*, *NUF2*, *TRIM59*, *FRZB*, *E2F8*, *TREM2*, and *MTHFD1L*. Additionally, 7 downregulated *PCK1*, *TSC22D3*, *SLC22A7*, *CYP2U1*, *SARDH*, *TTC36*, and *APOC1* were related to a worse overall survival rate in HCC patients suggesting their tumor suppressive effects. The known functions of these transcripts are summarized in Table 1. Thus, our findings, produced in mice, most of which did not have liver cancer, have human liver cancer relevance.

**Table 1:** Transcripts that are commonly changed by WD, aging, and FXR KO are associated with the overall survival rate in HCC patients (*p* value < 0.05). The “high” and “low” expression was defined as the upper and lower quartiles of expression for each gene (*n* = 108, 50% as a cutoff for both lower and upper quartiles)

Gene	Full Name	HCC *vs*. Normal WD *vs*. CD 15 *vs*. 5 FXR KO *vs*. WT	Association	Function
*PLEKHH1*	pleckstrin homology, MyTH4 and FERM domain containing H1	Up	Unfavored	Unknown
*TTC39A*	tetratricopeptide repeat domain 39A	Up	Unfavored	Unknown
*ATP6V0D2*	ATPase H+ transporting V0 subunit d2	Up	Unfavored	It is a subunit of the integral membrane V0 complex of vacuolar ATPase which is responsible for acidifying a variety of intracellular compartments in eukaryotic cells, thus providing most of the energy required for transport processes in the vacuolar system. Regulator of osteoclast fusion and bone formation
*CENPE*	centrosome-associated protein E	Up	Unfavored	Microtubule motor activity
*KIF20A*	kinesin family member 20A	Up	Unfavored	Microtubule motor activity
*ASPM*	abnormal spindle microtubule assembly	Up	Unfavored	Involved in mitotic spindle regulation and coordination of mitotic processes ^(36)^
*CKAP2L*	cytoskeleton associated protein 2 like	Up	Unfavored	Microtubule-associated protein required for mitotic spindle formation and cell-cycle progression in neural progenitor cells
*HMMR*	hyaluronan mediated motility receptor	Up	Unfavored	Involved in cell motility ^(30)^
*ECT2*	epithelial cell transforming 2	Up	Unfavored	A guanine nucleotide exchange factor and transforming protein that is related to Rho-specific exchange factors and yeast cell cycle regulators ^(34)^
*TOP2A*	DNA topoisomerase II alpha	Up	Unfavored	A DNA topoisomerase, an enzyme that controls and alters the topologic states of DNA during transcription ^(33)^
*KIF18B*	kinesin family member 18B	Up	Unfavored	Mitotic cell cycle; Microtubule motor activity; Protein binding; ATP binding; Kinesin binding ^(32)^
*TPX2*	TPX2 microtubule nucleation factor	Up	Unfavored	Apoptotic process; Mitotic nuclear division; Cell proliferation; Activation of protein kinase activity; Regulation of Mitotic spindle organization ^(28)^
*NUF2*	NUF2 component of NDC80 kinetochore complex	Up	Unfavored	A component of a conserved protein complex associated with the centromere ^(31)^
*TRIM59*	tripartite motif containing 59	Up	Unfavored	Negative regulation of I-kappaB kinase/NF-kappaB signaling ^(27)^
*FRZB*	frizzled related protein	Up	Unfavored	Negative regulation of cell proliferation; Negative regulation of the Wnt signaling pathway
*E2F8*	E2F transcription factor 8	Up	Unfavored	Regulates progression from G1 to S phase by ensuring the nucleus divides at the proper time ^(35)^
*TREM2*	triggering receptor expressed on myeloid cells 2	Up	Unfavored	Functions in immune response and may be involved in chronic inflammation by triggering the production of constitutive inflammatory cytokines
*MTHFD1L*	methylenetetrahydrofolate dehydrogenase (NADP+ dependent) 1 like	Up	Unfavored	Involved in the synthesis of tetrahydrofolate (THF) in the mitochondrion. THF is important in the de novo synthesis of purines and thymidylate and in the regeneration of methionine from homocysteine.
*PCK1*	phosphoenolpyruvate carboxykinase 1	Down	Favored	A main control point for the regulation of gluconeogenesis ^(37)^
*TSC22D3*	TSC22 domain family member 3	Down	Favored	Function as transcriptional regulators in the anti-inflammatory and immunosuppressive effects of this steroid and chemokine
*SLC22A7*	solute carrier family 22 member 7	Down	Favored	Involved in the sodium-independent transport and excretion of organic anions, some of which are potentially toxic.
*CYP2U1*	cytochrome p450 family 2 subfamily U member 1	Down	Favored	Xenobiotic metabolic process; Cell death; Arachidonic acid metabolic process
*SARDH*	sarcosine dehydrogenase	Down	Favored	An enzyme localized to the mitochondrial matrix which catalyzes the oxidative demethylation of sarcosine
*TTC36*	tetratricopeptide repeat domain 36	Down	Favored	May function as a tumor suppressor in hepatocellular carcinoma (HCC) since it promotes apoptosis but is downregulated in HCC.
*APOC1*	apolipoprotein C1	Down	Favored	High-density lipoprotein and very low-density lipoprotein metabolism; Inhibits cholesteryl ester transfer protein in plasma

Superscript is the information of references related to human HCC

Additionally, 44 hepatic metabolites (e.g., leucine, urea, ribitol, palmitic acid, oxalic acid, myo-inositol, succinic acid, ethanolamine, uracil, sorbitol, galactitol, and taurine) were commonly altered by WD, aging, and FXR deactivation (Fig. S20). To further identify liver disease-related metabolites or gut microbes, an association analysis was performed between hepatic features (76 transcripts and 44 metabolites) and non-hepatic features (serum/urine metabolites and cecal microbiota at genus level) (Fig. S21). An expanded heatmap revealed the expressions of those 25 hepatic transcripts, which predict human HCC survival (Table 1), correlated with serum or urine metabolites and microbes (Fig. S21). The 18 genes whose upregulation predicts worse survival of HCC patients (red) were positively correlated with serum N-methylhydantoin and ornithine, as well as cecal *Dehalobacterium*, *Bacteroides*, and *Desulfovibrio.* The 7 genes whose downregulation predicts worse survival of HCC patients (blue) were positively associated with serum concentrations of formate, arginine, betaine, and glycolate.

## Discussion

This study investigated the impact of WD intake and aging on metabolic liver disease development in WT and FXR KO mice based on multi-omics data. It revealed the indispensable roles of FXR in regulating age and diet-associated liver metabolism. Common and unique hepatic transcripts, liver, serum, and urine metabolites, as well as cecal microbiota affected by diet, and/or age, and/or FXR deactivation were uncovered to identify metabolic features for the healthy liver, NAFLD, and HCC. Transcripts and metabolites as well as their pathways in the liver represent hepatic phenotypes induced by WD, aging, and FXR deactivation. Metabolomics of serum and urine and gut microbiota reflect patterns of metabolic dysfunction, which can guide diagnosis and improve insight into the pathophysiology of metabolic liver diseases.

WD-fed WT mice have metabolic perturbations with mild NASH [22]. Consistently, transcriptomic data revealed that the cholesterol biosynthesis pathway was inhibited, whereas genes involved in the regulation of cytokine production were upregulated by WD intake. We previously found that prolonged WD intake led to hepatic lymphocyte and neutrophil infiltration when mice reached 10 months of age [5]. Furthermore, many proinflammatory cytokine and chemokine genes including the *Ccl17*, *Ccl20*, *Ccl2*, *Tnf*, and *Il6* were induced in 15-month-old mice revealing the combined impact of diet and age [4]. Reduced OXPHOS in WD-fed WT mice, which indicates mitochondrial dysfunction, together with heightened inflammation contribute to deregulated lipid and glucose metabolism, insulin resistance, and cancer development [23–25].

In FXR KO mice, WD intake increased serum alanine aminotransferase at 5 months of age indicating liver injury [14]. WD uniquely enhanced the expression of iron binding related genes in FXR KO mice. Iron overload leads to liver injury through the production of reactive oxygen species, which is implicated in liver carcinogenesis [26]. However, the exact role of FXR in regulating iron remains to be studied.

PCA of hepatic transcriptomics data revealed that WD intake facilitated aging, which is characterized by inflammageing. In consistency, 15-month-old livers had induced cytokine, inflammatory responses, leukocyte migration, myeloid leukocyte activation, etc. Moreover, similar to WD intake, many age-related changes in hepatic transcriptomes found in WT mice were no longer noted in FXR KO mice. Notably, OXPHOS, the process to generate ATP in mitochondria, was the top downregulated pathway caused by WD intake as well as aging in an FXR-dependent manner. Along the same line, 15-month-old livers had reduced mitochondrial transmembrane transport, energy coupled proton transport, and mitochondrion organization at the transcript level. Together, activation of FXR alleviates aging and diet-reduced OXPHOS at molecular levels.

By examining the differential expression of hepatic genes between WT and FXR KO mice, the specific roles of FXR became apparent. Irrespective of diets, FXR inactivation increased inflammatory responses but inhibited the fatty acid metabolic process. In healthy mice, FXR also uniquely dictates neuron differentiation, muscle contraction, organ growth, and actin cytoskeleton organization in addition to metabolism. Moreover, WD intake further enriched the chemical carcinogenesis, extracellular matrix, and blood vessel development pathways in FXR KO mice leading to tumorigenesis. A limitation of the current study is that those novel roles of FXR found at molecular levels remained to be validated at phenotypic levels using other animal models.

The commonality of diet, age, and FXR KO was uncovered in the current study. The data revealed 654 hepatic transcripts commonly altered with differential expressions due to diet intake, age, and FXR deficiency. Those transcripts have the potential to be metabolic disease development signatures. Among them, 76 transcripts have been documented to be differentially expressed in human HCC vs. healthy livers. Moreover, 25 out of those 76 transcripts can predict the overall survival rate of HCC patients (Table 1). Moreover, transcripts *Trim59*, *Tpx2*, *Kif20a*, *Hmmr*, and *Nuf2* are known biomarkers of human HCC [27–31]. Additionally, upregulations of *Kif18b*, *Top2a*, *Ect2*, *E2f8*, and *Aspm* promote human HCC progression [32–36]. In contrast, *Pck1*, *Tsc22d3*, *Ttc36*, *Slc22a7*, *Cyp2u1*, *Sardh*, and *Apoc1*, whose expressions are downregulated due to WD intake, aging, and FXR KO, are reduced in human HCC. Furthermore, overexpression of the *Pck1* gene protects against HCC via activating gluconeogenesis and inhibiting glycolysis pathways [37]. These observations revealed the relevance of our data to human HCC. Together, the generated data uncovered additional hundreds of biomarkers that may be potential early markers for HCC, which remain to be validated using human specimens.

The commonality of diet, age, and FXR KO was uncovered in the current study. The data revealed 654 hepatic transcripts commonly altered with differential expressions due to diet intake, age, and FXR deficiency. Those transcripts have the potential to be metabolic disease development signatures. Among them, 76 transcripts have been documented to be differentially expressed in human HCC vs. healthy livers. Moreover, 25 out of those 76 transcripts can predict the overall survival rate of HCC patients (Table 1). Moreover, transcripts *Trim59*, *Tpx2*, *Kif20a*, *Hmmr*, and *Nuf2* are known biomarkers of human HCC [27–31]. Additionally, upregulations of *Kif18b*, *Top2a*, *Ect2*, *E2f8*, and *Aspm* promote human HCC progression [32–36]. In contrast, *Pck1*, *Tsc22d3*, *Ttc36*, *Slc22a7*, *Cyp2u1*, *Sardh*, and *Apoc1*, whose expressions are downregulated due to WD intake, aging, and FXR KO, are reduced in human HCC. Furthermore, overexpression of the *Pck1* gene protects against HCC via activating gluconeogenesis and inhibiting glycolysis pathways [37]. These observations revealed the relevance of our data to human HCC. Together, the generated data uncovered additional hundreds of biomarkers that may be potential early markers for HCC, which remain to be validated using human specimens.

Metabolites can impact hepatic inflammation and metabolic disease development. In the liver, irrespective of age, WD increased uracil, oxoproline, and myo-inositol but decreased linoleic acid. Uracil and oxoproline (a cyclized derivative of L-glutamic acid) are etiologic factors for NASH [38]. Myo-inositol is a sugar alcohol and a precursor of inositol triphosphate, acting as an intracellular second messenger and regulating hormonal signaling including insulin [39, 40]. In consistency, linoleic acid is decreased in aging mice and human HCC [41]. In addition, WD enriched steroid biosynthesis with increased squalene in WT mice, and the accumulation of squalene in the liver decreases hepatic cholesterol and triglycerides [42]. Citrate cycle/TCA cycle and glycolysis/gluconeogenesis, enriched by WD only in FXR KO mice, are associated with type 2 diabetes [43].

Hepatic alanine, aspartate, and glutamate metabolism is one of the most significant pathways altered by aging in WT but not in FXR KO mice. It is also the top pathway deregulated by FXR deactivation. Those amino acid metabolic pathways might be involved in the pathogenesis of metabolic syndrome [44]. In addition, aging decreased ethanolamine (amino alcohol) and metabolites involved in the aminoacyl-tRNA biosynthesis in WT mice. Ethanolamine is a precursor for phospholipids synthesis. Dietary ethanolamine exhibits a protective effect against hyperlipidemia in aged mice [45]. Reduced hepatic ethanolamine was consistently found in WD-fed WT mice further suggesting its protective role. Moreover, ursodeoxycholic acid, which was reduced by WD and FXR KO, has hepatoprotective effects partly by regulating the aminoacyl-tRNA biosynthesis [5, 46]. Thus, at the molecular level, hepatic amino acid metabolism in aging is dependent on FXR.

By comparing FXR KO with WT mouse livers, FXR is essential for propanoate metabolism (reduced 2-hydroxybutyric acid, increased 2-ketobutyric acid, succinic acid, and beta-alanine). 2-Hydroxybutyric acid is produced by threonine and methionine as well as glutathione anabolism. It metabolizes into propionyl-CoA, a coenzyme A derivative of propionic acid, which converts into succinyl CoA and participates in the TCA cycle and gluconeogenesis. 2-Hydroxybutyric acid is functionally related to butyric acid which is associated with gut microbiota, and vancomycin pretreatment increases serum 2-hydroxybutyric acid [47]. It has been shown that 2-hydroxybutyric acid via intraperitoneal injection protects against acetaminophen-induced liver injury in mice [47]. In contrast, succinic acid has been identified as an oncometabolite in causing cancer [48]. Consistently, the highest concentration of hepatic succinic acid was observed in WD-fed FXR KO at 15 months of age.

FXR KO reduced hepatic melibiose in a diet-independent manner. Melibiose is a nondigestible disaccharide formed by an α-1,6 linkage between galactose and glucose. Cleaved by α-galactosidase found in *Saccharomyces pastorianus*, melibiose breaks down into glucose and galactose. Decreased hepatic melibiose in FXR KO mice reveals the significance of FXR in regulation of sugar metabolism via the gut-liver axis. Specific to WD-fed animals, FXR KO induces hepatic orotic acid and 3-phosphoglycerate, which have been implicated in tumorigenesis [49, 50].

It is interesting to note that altered hepatic arginine biosynthesis is shared by dietary intervention, aging, and FXR KO. Arginine is synthesized from citrulline in the urea cycle, which is an energetically costly process, and further metabolized into urea. Increased urea was consistently found in the livers of aged mice. Urea is derived from ammonia, which is generally considered to play a role in hepatic encephalopathy [51]. The potential roles of the ammonia-urea cycle in metabolic disease development and liver injury warrant further investigation.

Hepatic fumaric acid (dicarboxylic acid) involved in arginine biosynthesis was increased by both WD and FXR KO. Fumaric acid is formed by the oxidation of succinic acid and a precursor of L-malate in the TCA cycle. In consistency, malic acid was also elevated in WD-fed FXR KO mice. Fumaric acid has been identified as a cancer-causing metabolite [52], indicating TCA cycle involves in the pathogenetic arginine biosynthesis during metabolic liver disease development (Fig. S18).

Serum metabolomics can distinguish chronological ages in both genotypes. Aging increased amino acids (alanine, isoleucine) but decreased 1.3-dihydroxyacetone (DHA, non-toxic sugar) and ketone bodies (acetone, acetoacetate) in the serum. In consistency, hepatic alanine and isoleucine also increased with age. Alanine is produced from pyruvate by transamination. Isoleucine is a branch-chain amino acid (BCAA), and circulating BCAA increases in humans with metabolic diseases including obesity and type 2 diabetes [53].

In contrast with serum metabolome, urine metabolome distinguished dietary effects in both genotypes based on PCA. There was no apparent kidney injury in any of those studied mice. As signatures of WD intake, urine TMA and trimethylamine N-oxide (TMAO) were reduced. Additionally, FXR KO also reduced urine TMA. TMAO is synthesized endogenously from TMA, which is generated from microbial metabolism of choline, lecithin, or carnitine. TMA via the portal circulation converts into TMAO in the liver. A high-fat diet can reduce the ratio of TMA to TMAO in the urine, and TMAO supplementation aggravates liver steatosis by inhibiting FXR [15, 54].

Knockout of FXR also leads to metabolic deterioration based on urine metabolomics. FXR KO increased urine 2-hydroxyvalerate, which is increased with lactic acidosis occurring in succinic acidemia [55]. In line, hepatic succinic acid was elevated in FXR KO mice which are cancer prone. In contrast, FXR KO reduced urine taurine and creatinine. Taurine is an essential amino acid that conjugates bile acids. The reduced output might suggest increased demand in conjugation. Creatinine is a waste product and its reduction in urine might be due to inefficient kidney/glomerular filtration.

PCA showed that the cecal microbiota community structure was distinctly different based on ages dependent on FXR. *Erysipelotrichaceae* and *Lachnospiraceae* (Fig. S12), as butyrate-producing bacteria, were reduced in aged WT mice. Age associated reduction of *Rikenellaceae* was only found in the absence of FXR, and the reduced *Rikenellaceae* is also found in NAFLD [56]. FXR KO mice also have a distinct gut microbiota structure in comparison to the WT, and *Bacteroidaceae* are expanded in FXR KO mice [4]. We further showed that *Bacteroides* at genus level under the *Bacteroidaceae* family was consistently increased due to lack of FXR irrespective of diets and ages.

Using data generated from non-liver specimens, association analysis (Fig. S21) revealed that serum betaine reduction was linked with the elevated expression of genes implicated in HCC poor survival. Betaine is an antioxidant that inhibits inflammation and apoptosis but upregulates cytoprotective Akt/mTOR signaling in fatty liver disease [57]. In this study, serum betaine was reduced in FXR-deficient mice. Moreover, FXR KO increased the relative abundance of *Dehalobacterium*, *Bacteroides,* and *Desulfovibrio*, which were linked with high expression levels of genes implicated in HCC poor survival. It has been shown that these three expanded bacteria contribute to metabolic liver disease development in the mouse model [58, 59]. Fecal metabolites were not analyzed in the current study. Others omics data such as lipidomics and glycomics were also not done. Additional analyses will uncover other biomarkers for the risks of liver metabolic disease development including NASH and HCC.

## Conclusions

Collectively, FXR can be a target for improving human HCC survival, and metabolites or bacteria from noninvasive specimens can potentially be used for the early detection markers of metabolic liver diseases.

## Supplementary Information

**Additional file 1: Fig. S1.** Serum cholesterol and triglycerides levels in WT and FXR KO mice fed with either a CD or WD in 3 age groups. (A) Serum cholesterol levels (Compared with 5-month-old CD-WT, * *p* < 0.05, ** *p* < 0.01, *** *p* < 0.001) (B) Serum triglycerides levels (Compared with 5-month-old CD-WT, ^#^ *p* < 0.05, ^##^ *p* < 0.01, ^###^ *p* < 0.001). Unpaired t-test.

**Additional file 2: Fig. S2.** Heatmaps show the fold changes of WD-altered 36 transcripts in (A) WT mice and 6 transcripts in (B) FXR KO mice regardless of ages (fold change ≥2 and adjusted *p* value < 0.05). **Fig. S3.** Altered liver metabolites due to differential dietary intake. (A) Diet altered metabolites in both WT and FXR KO mice. (B) Diet changed metabolites only in WT or FXR KO mice (raw *p* value < 0.05 and FDR < 0.1). **Fig. S4.** The effects of diets on hepatic bile acids in WT and FXR KO mice. (A) Principal component analyses of hepatic bile acids of WT and FXR KO mice fed with either a CD or WD. (B) Venn diagrams show the numbers of distinct and overlapping bile acids that were changed by differential diets intake in 3 age groups (*p* < 0.05). (C) A heatmap of relative concentrations of hepatic bile acids. **Fig. S5.** The effects of diets on serum metabolomes in WT and FXR KO mice. (A) Principal component analyses of serum metabolomes of WT and FXR KO mice fed with either a CD or WD. (B) Venn diagrams show the numbers of distinct and overlapping metabolites that were changed by differential diets intake in 3 age groups (raw *p* value < 0.05 and FDR < 0.1). The metabolites in purple were affected by diet in both genotypes. **Fig. S6.** The effects of diets on urine metabolomes in WT and FXR KO mice. (A) Principal component analyses of urine metabolomes of WT and FXR KO mice. (B) Venn diagrams show the numbers of distinct and overlapping metabolites that were changed due to differential diet intake in 3 age groups (raw *p* value < 0.05 and FDR < 0.1). The metabolites in purple are commonly affected by diet in both genotypes. **Fig. S7.** The effects of diets on cecal microbiota. (A) Principal component analyses of cecal microbiota at genus level of WT and FXR KO mice fed with either a CD or WD. (B) Venn diagrams show the numbers of distinct and overlapping cecal microbiota at the genus level that were changed by differential diets intake in 3 age groups (raw *p* value < 0.05). **Fig. S8.** Age altered liver metabolites in WT and FXR KO mice. (A) Heatmap of metabolites that changed due to age (15 vs. 5) shared in both WT and FXR KO mice. (B) Age-changed metabolites in WT or FXR KO mice (raw *p* value < 0.05 and FDR < 0.1). **Fig. S9.** The influence of ages on hepatic bile acids in WT and FXR KO mice. (A) Principal component analyses of hepatic bile acids of 5- or 15-month-old WT and FXR KO mice fed with either a CD or WD. (B) Venn diagrams show the numbers of distinct and overlapping bile acids that were changed in two age groups. **Fig. S10.** The influence of ages on serum metabolomes in WT and FXR KO mice. (A) Principal component analyses of serum metabolomes of 5- or 15-month-old WT and FXR KO mice fed with either a CD or WD. (B) Venn diagrams show the numbers of distinct and overlapping metabolites that were changed in two age groups. The metabolites in purple were commonly affected by ages in both genotypes (raw *p* value < 0.05 and FDR < 0.1). **Fig. S11.** The influence of ages on urine metabolomes in WT and FXR KO mice. (A) Principal component analyses of urine metabolomes of 5- or 15-month-old WT and FXR KO mice fed with either a CD or WD. (B) Venn diagrams show the numbers of distinct and overlapping metabolites that were changed by age (raw *p* value < 0.05 and FDR < 0.1). The metabolites in purple are commonly affected by age in both genotypes. **Fig. S12.** The influence of ages on cecal microbiota in WT and FXR KO mice. (A) Principal component analyses of cecal microbiota at genus level of 5- or 15-month-old WT and FXR KO mice fed with either a CD or WD . (B) Venn diagrams show the numbers of distinct and overlapping bacteria at the genus level that were changed by age (raw *p* value < 0.05). **Fig. S13.** Altered liver metabolites due to FXR KO. (A) A heatmap shows FXR KO-altered metabolites commonly found in mice with either a CD or WD. (B) FXR KO changed metabolites in WT or FXR KO mice (raw *p* value < 0.05 and FDR < 0.1). **Fig. S14.** The effects of FXR KO on hepatic bile acids. (A) Principal component analyses of serum metabolomes of WT and FXR KO mice fed with either a CD or WD and euthanized when they were 5, 10, and 15 months old. (B) Venn diagrams show the numbers of distinct and overlapping metabolites that were altered by FXR KO in CD- and WD-fed mice (raw p value < 0.05 and FDR < 0.1). **Fig. S15.** The effects of FXR KO on serum metabolomes. (A) Principal component analyses of serum metabolomes of WT and FXR KO mice fed with either a CD or WD and euthanized when they were 5, 10, and 15 months old. (B) Venn diagrams show the numbers of distinct and overlapping metabolites that were altered by FXR KO in CD- and WD-fed mice (raw *p* value < 0.05 and FDR < 0.1). The metabolites in purple were commonly changed by FXR KO in both CD- and WD-fed mice. **Fig. S16.** A heatmap shows serum metabolite levels in WT and FXR KO mice fed with either a CD or WD for different durations. **Fig. S17.** The effects of FXR KO on urine metabolomes. (A) Principal component analyses of urine metabolomes of WT and FXR KO mice fed with either a CD or WD and euthanized when mice were 5, 10, and 15 months old. (B) Venn diagrams show the numbers of distinct and overlapping metabolites that were altered by FXR KO in CD- and WD-fed mice (raw *p* value < 0.05 and FDR < 0.1). The metabolites in purple were commonly changed by FXR KO in both CD- and WD-fed mice. **Fig. S18.** A heatmap shows urine metabolite levels in WT and FXR KO mice fed with either a CD or WD for different durations. **Fig. S19.** The effects of FXR KO on cecal microbiota. (A) Principal component analyses of cecal microbiota at genus level of WT and FXR KO mice fed with either a CD or WD and euthanized when mice were 5, 10, and 15 months old. (B) Venn diagrams show the numbers of distinct and overlapping bacteria that were altered by FXR KO in CD- and WD-fed mice. The bacteria in purple were commonly changed by FXR KO in both CD- and WD-fed mice. (C) A heatmap of relative abundances of cecal microbiota at genus level that were changed by FXR KO irrespective of ages (raw *p* value < 0.05). **Fig. S20.** Common changes in hepatic metabolites due to WD intake, aging, and FXR KO. Venn diagram shows the number of altered metabolites by each risk factor. Heatmap of 44 hepatic metabolites that were commonly altered by diet, age, and FXR KO (raw *p* value < 0.05 and FDR < 0.1). **Fig. S21.** Spearman’s correlation analysis between hepatic features (76 transcripts and 44 metabolites that were commonly altered by diet, age, and FXR KO) and serum/urine metabolites as well as cecal microbiota at the genus level. *p < 0.05, **p < 0.01 with Hochberg correction. Upregulated transcripts are in red, while downregulated ones are in blue. **Fig. S22.** Diet, age, and FXR KO altered hepatic, serum, and urine metabolites involved in the urea cycle, TCA cycle, and methionine cycle.

**Additional file 3: Table S1.** The function of hepatic transcripts that were consistently altered by WD intake in WT and FXR KO mice irrespective of their ages.

**Additional file 4: Table S2.** RNA sequence quality data.

## Data Availability

RNA sequencing data are available on Gene Expression Omnibus (https://www.ncbi.nlm.nih.gov/geo/) (GSE216375). All data to support the conclusions are present in the paper and supplemental Figures/Tables. Additional information related to this paper can be requested from the authors.

## Abbreviations

BA: Bile acid
CD: Control diet
DEGs: Differentially expressed genes
FXR: Farnesoid X receptor
GPBAR1: G protein-coupled bile acid receptor 1
HCC: Hepatocellular carcinoma
KO: Knockout
NAFLD: non-alcoholic fatty liver disease
NASH: Non-alcoholic steatohepatitis
OXPHOS: Oxidative phosphorylation
PCA: Principal component analysis
TCGA: The Cancer Genome Atlas
TMA: Trimethylamine
TMAO: Trimethylamine N-oxide
WD: Western diet
WT: Wild-type

## References

[1] Ioannou GN (2021). Epidemiology and risk-stratification of NAFLD-associated HCC. J Hepatol.

[2] Juanola O, Martínez-López S, Francés R, Gómez-Hurtado I. Non-alcoholic fatty liver disease: metabolic, genetic, epigenetic and environmental risk factors. Int J Environ Res Public Health. 2021;18(10):5227. 10.3390/ijerph18105227 PMC8155932 34069012

[3] Sheng L, Jena PK, Hu Y, Wan YY (2021). Age-specific microbiota in altering host inflammatory and metabolic signaling as well as metabolome based on the sex. Hepatobiliary Surg Nutr.

[4] Sheng L, Jena PK, Hu Y, Liu HX, Nagar N, Kalanetra KM (2017). Hepatic inflammation caused by dysregulated bile acid synthesis is reversible by butyrate supplementation. J Pathol.

[5] Jena PK, Sheng L, Liu HX, Kalanetra KM, Mirsoian A, Murphy WJ (2017). Western diet-induced dysbiosis in farnesoid X receptor knockout mice causes persistent hepatic inflammation after antibiotic treatment. Am J Pathol.

[6] Jena PK, Sheng L, Li Y, Wan YY (2020). Probiotics VSL#3 are effective in reversing non-alcoholic steatohepatitis in a mouse model. Hepatobiliary Surg Nutr.

[7] Jena PK, Sheng L, Nguyen M, Di Lucente J, Hu Y, Li Y (2020). Dysregulated bile acid receptor-mediated signaling and IL-17A induction are implicated in diet-associated hepatic health and cognitive function. Biomark Res.

[8] Xiong X, Wang X, Lu Y, Wang E, Zhang Z, Yang J (2014). Hepatic steatosis exacerbated by endoplasmic reticulum stress-mediated downregulation of FXR in aging mice. J Hepatol.

[9] Lefebvre P, Staels B (2014). Failing FXR expression in the liver links aging to hepatic steatosis. J Hepatol.

[10] Wu L, Feng J, Li J, Yu Q, Ji J, Wu J (2021). The gut microbiome-bile acid axis in hepatocarcinogenesis. Biomedi Pharmacother= Biomedecine & pharmacotherapie.

[11] Huang X-f, Zhao W-y, Huang W-d (2015). FXR and liver carcinogenesis. Acta Pharmacol Sin.

[12] Kim I, Morimura K, Shah Y, Yang Q, Ward JM, Gonzalez FJ (2007). Spontaneous hepatocarcinogenesis in farnesoid X receptor-null mice. Carcinogenesis.

[13] Su H, Ma C, Liu J, Li N, Gao M, Huang A (2012). Downregulation of nuclear receptor FXR is associated with multiple malignant clinicopathological characteristics in human hepatocellular carcinoma. Am J Physiol Gastrointest Liver Physiol.

[14] Sheng L, Jena PK, Liu HX, Kalanetra KM, Gonzalez FJ, French SW (2017). Gender differences in bile acids and microbiota in relationship with gender dissimilarity in steatosis induced by diet and FXR inactivation. Sci Rep.

[15] Hasegawa Y, Chen S-Y, Sheng L, Jena PK, Kalanetra KM, Mills DA (2020). Long-term effects of western diet consumption in male and female mice. Sci Rep.

[16] Sinal CJ, Tohkin M, Miyata M, Ward JM, Lambert G, Gonzalez FJ (2000). Targeted disruption of the nuclear receptor FXR/BAR impairs bile acid and lipid homeostasis. Cell.

[17] Patro R, Duggal G, Love MI, Irizarry RA, Kingsford C (2017). Salmon provides fast and bias-aware quantification of transcript expression. Nat Methods.

[18] Soneson C, Love MI, Robinson MD. Differential analyses for RNA-seq: transcript-level estimates improve gene-level inferences. F1000Res. 2015;4:1521. 10.12688/f1000research.7563.1 PMC4712774 26925227

[19] Zhu A, Ibrahim JG, Love MI (2019). Heavy-tailed prior distributions for sequence count data: removing the noise and preserving large differences. Bioinformatics (Oxford, England).

[20] Subramanian A, Tamayo P, Mootha VK, Mukherjee S, Ebert BL, Gillette MA (2005). Gene set enrichment analysis: a knowledge-based approach for interpreting genome-wide expression profiles. Proc Natl Acad Sci U S A.

[21] Dubois-Chevalier J, Dubois V, Dehondt H, Mazrooei P, Mazuy C, Sérandour AA (2017). The logic of transcriptional regulator recruitment architecture at cis-regulatory modules controlling liver functions. Genome Res.

[22] Machado MV, Michelotti GA, Xie G, de Almeida TP, Boursier J, Bohnic B (2015). Mouse models of diet-induced nonalcoholic steatohepatitis reproduce the heterogeneity of the human disease. PLoS One.

[23] Kim J-a, Wei Y, Sowers JR (2008). Role of mitochondrial dysfunction in insulin resistance. Circ Res.

[24] Ribas V, García-Ruiz C, Fernández-Checa JC (2016). Mitochondria, cholesterol and cancer cell metabolism. ClinTransl Med.

[25] Bournat JC, Brown CW (2010). Mitochondrial dysfunction in obesity. Curr Opin Endocrinol Diabetes Obes.

[26] Miyanishi K, Tanaka S, Sakamoto H, Kato J (2019). The role of iron in hepatic inflammation and hepatocellular carcinoma. Free Radic Biol Med.

[27] Khatamianfar V, Valiyeva F, Rennie PS, Lu WY, Yang BB, Bauman GS (2012). TRIM59, a novel multiple cancer biomarker for immunohistochemical detection of tumorigenesis. BMJ Open.

[28] Neumayer G, Belzil C, Gruss OJ, Nguyen MD (2014). TPX2: of spindle assembly, DNA damage response, and cancer. Cell Mol Life Sci.

[29] Li L, Lei Q, Zhang S, Kong L, Qin B (2017). Screening and identification of key biomarkers in hepatocellular carcinoma: evidence from bioinformatic analysis. Oncol Rep.

[30] Guo H, Fan Q (2021). Identification of the HMMR gene as a diagnostic and prognostic biomarker in hepatocellular carcinoma based on integrated bioinformatics analysis. Evid Based Complement Alternat Med.

[31] Xie X, Jiang S, Li X (2021). Nuf2 is a prognostic-related biomarker and correlated with immune infiltrates in hepatocellular carcinoma. Front Oncol.

[32] Yang B, Wang S, Xie H, Wang C, Gao X, Rong Y (2020). KIF18B promotes hepatocellular carcinoma progression through activating Wnt/β-catenin-signaling pathway. J Cell Physiol.

[33] Wang T, Lu J, Wang R, Cao W, Xu J (2022). TOP2A promotes proliferation and metastasis of hepatocellular carcinoma regulated by miR-144-3p. J Cancer.

[34] Xu D, Wang Y, Wu J, Zhang Z, Chen J, Xie M (2021). ECT2 overexpression promotes the polarization of tumor-associated macrophages in hepatocellular carcinoma via the ECT2/PLK1/PTEN pathway. Cell Death Dis.

[35] Lv Y, Xiao J, Liu J, Xing F (2017). E2F8 is a potential therapeutic target for hepatocellular carcinoma. J Cancer.

[36] Zhang H, Yang X, Zhu L, Li Z, Zuo P, Wang P (2021). ASPM promotes hepatocellular carcinoma progression by activating Wnt/β-catenin signaling through antagonizing autophagy-mediated Dvl2 degradation. FEBS Open Bio.

[37] Tang Y, Zhang Y, Wang C, Sun Z, Li L, Cheng S (2018). Overexpression of PCK1 gene antagonizes hepatocellular carcinoma through the activation of gluconeogenesis and suppression of glycolysis pathways. Cell Physiol Biochem.

[38] Qi S, Xu D, Li Q, Xie N, Xia J, Huo Q (2017). Metabonomics screening of serum identifies pyroglutamate as a diagnostic biomarker for nonalcoholic steatohepatitis. Clin Chim Acta.

[39] Croze ML, Soulage CO (2013). Potential role and therapeutic interests of myo-inositol in metabolic diseases. Biochimie.

[40] Chhetri DR (2019). Myo-inositol and its derivatives: their emerging role in the treatment of human diseases. Front Pharmacol.

[41] Ferrarini A, Di Poto C, He S, Tu C, Varghese RS, Kara Balla A (2019). Metabolomic analysis of liver tissues for characterization of hepatocellular carcinoma. J Proteome Res.

[42] Lou-Bonafonte JM, Martínez-Beamonte R, Sanclemente T, Surra JC, Herrera-Marcos LV, Sanchez-Marco J, et al. Current insights into the biological action of squalene. Mol Nutr Food Res. 2018;62(15):e1800136. 10.1002/mnfr.201800136 29883523

[43] Guasch-Ferré M, Santos JL, Martínez-González MA, Clish CB, Razquin C, Wang D (2020). Glycolysis/gluconeogenesis- and tricarboxylic acid cycle-related metabolites, Mediterranean diet, and type 2 diabetes. Am J Clin Nutr.

[44] Sookoian S, Pirola CJ (2012). Alanine and aspartate aminotransferase and glutamine-cycling pathway: their roles in pathogenesis of metabolic syndrome. World J Gastroenterol.

[45] Ding L, Zhang L, Shi H, Xue C, Yanagita T, Zhang T (2020). The protective effect of dietary EPA-enriched ethanolamine plasmalogens against hyperlipidemia in aged mice. Eur J Lipid Sci Technol.

[46] Kim DJ, Chung H, Ji SC, Lee S, Yu KS, Jang IJ (2019). Ursodeoxycholic acid exerts hepatoprotective effects by regulating amino acid, flavonoid, and fatty acid metabolic pathways. Metabolomics.

[47] Zheng N, Gu Y, Hong Y, Sheng L, Chen L, Zhang F (2020). Vancomycin pretreatment attenuates acetaminophen-induced liver injury through 2-hydroxybutyric acid. J Pharm Anal.

[48] Xia L, Zhang H, Wang X, Zhang X, Nie K (2021). The role of succinic acid metabolism in ovarian cancer. Front Oncol.

[49] Winter H, Kaisaki PJ, Harvey J, Giacopuzzi E, Ferla MP, Pentony MM, et al. Identification of circulating genomic and metabolic biomarkers in intrahepatic cholangiocarcinoma. Cancers (Basel). 2019;11(12):1895. 10.3390/cancers11121895 PMC6966597 31795195

[50] Li M, Wu C, Yang Y, Zheng M, Yu S, Wang J (2021). 3-phosphoglycerate dehydrogenase: a potential target for cancer treatment. Cell Oncol (Dordr).

[51] Parekh PJ, Balart LA (2015). Ammonia and its role in the pathogenesis of hepatic encephalopathy. Clin Liver Dis.

[52] Ni Y, Xie G, Jia W (2014). Metabonomics of human colorectal cancer: new approaches for early diagnosis and biomarker discovery. J Proteome Res.

[53] Lynch CJ, Adams SH (2014). Branched-chain amino acids in metabolic signalling and insulin resistance. Nat Rev Endocrinol.

[54] Tan X, Liu Y, Long J, Chen S, Liao G, Wu S (2019). Trimethylamine N-oxide aggravates liver steatosis through modulation of bile acid metabolism and inhibition of farnesoid x receptor signaling in nonalcoholic fatty liver disease. Mol Nutr Food Res.

[55] Asano K, Miyamoto I, Matsushita T, Murakami Y, Minoura S, Wagatsuma T (1988). Succinic acidemia: a new syndrome of organic acidemia associated with congenital lactic acidosis and decreased NADH-cytochrome c reductase activity. Clin Chim Acta.

[56] Michels N, Zouiouich S, Vanderbauwhede B, Vanacker J, Indave Ruiz BI, Huybrechts I (2022). Human microbiome and metabolic health: an overview of systematic reviews. Obes Rev.

[57] Veskovic M, Mladenovic D, Milenkovic M, Tosic J, Borozan S, Gopcevic K (2019). Betaine modulates oxidative stress, inflammation, apoptosis, autophagy, and Akt/mTOR signaling in methionine-choline deficiency-induced fatty liver disease. Eur J Pharmacol.

[58] Le HH, Lee MT, Besler KR, Johnson EL (2022). Host hepatic metabolism is modulated by gut microbiota-derived sphingolipids. Cell Host Microbe.

[59] Zhang X, Coker OO, Chu ES, Fu K, Lau HCH, Wang YX (2021). Dietary cholesterol drives fatty liver-associated liver cancer by modulating gut microbiota and metabolites. Gut.

