# Supplementary figures and images for: The essential roles of FXR in diet and age influenced metabolic changes and liver disease development: a multi-omics study
Source: Biomark Res. 2023 Feb 18;11:20. doi: 10.1186/s40364-023-00458-9 (PMC9938992; doi:10.1186/s40364-023-00458-9)

(A)

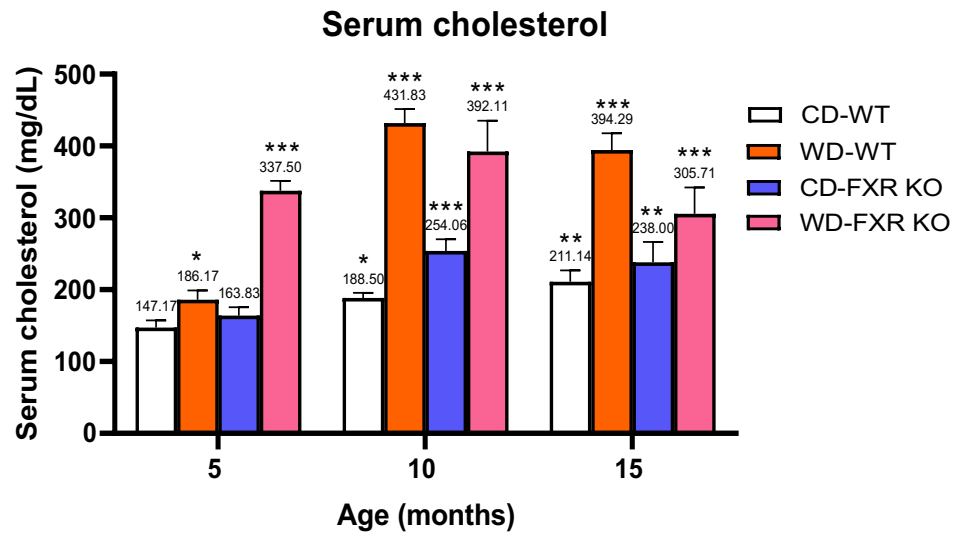

(B)

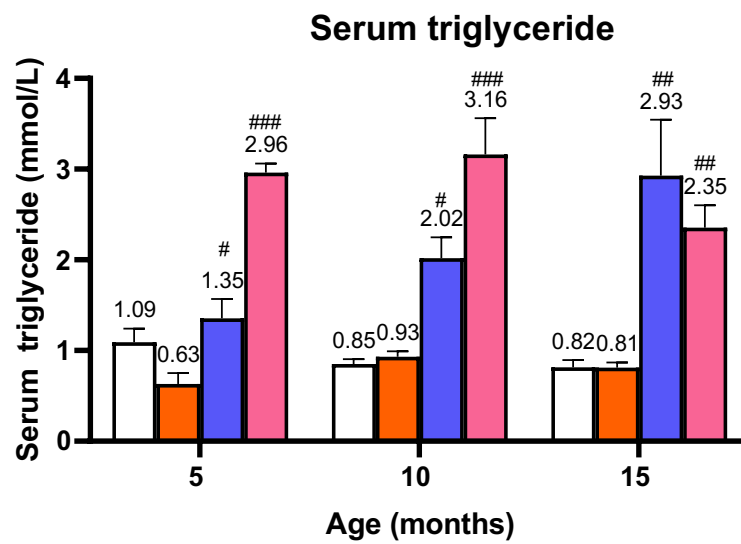

Supplement: Supplementary file 1 — Additional file 1: Fig. S1. Serum cholesterol and triglycerides levels in WT and FXR KO mice fed with either a CD or WD in 3 age groups. (A) Serum cholesterol levels (Compared with 5-month-old CD-WT, * p < 0.05, ** p < 0.01, *** p < 0.001) (B) Serum triglycerides levels (Compared with 5-month-old CD-WT, # p < 0.05, ## p < 0.01, ### p < 0.001). Unpaired t-test. [file 40364_2023_458_MOESM1_ESM.pdf]
