# Supplementary material for: The essential roles of FXR in diet and age influenced metabolic changes and liver disease development: a multi-omics study
Source: Biomark Res. 2023 Feb 18;11:20. doi: 10.1186/s40364-023-00458-9 (PMC9938992; doi:10.1186/s40364-023-00458-9)
Supplement: Supplementary file 2 — Additional file 2: Fig. S2. Heatmaps show the fold changes of WD-altered 36 transcripts in (A) WT mice and 6 transcripts in (B) FXR KO mice regardless of ages (fold change ≥2 and adjusted p value < 0.05). Fig. S3. Altered liver metabolites due to differential dietary intake. (A) Diet altered metabolites in both WT and FXR KO mice. (B) Diet changed metabolites only in WT or FXR KO mice (raw p value < 0.05 and FDR < 0.1). Fig. S4. The effects of diets on hepatic bile acids in WT and FXR KO mice. (A) Principal component analyses of hepatic bile acids of WT and FXR KO mice fed with either a CD or WD. (B) Venn diagrams show the numbers of distinct and overlapping bile acids that were changed by differential diets intake in 3 age groups (p < 0.05). (C) A heatmap of relative concentrations of hepatic bile acids. Fig. S5. The effects of diets on serum metabolomes in WT and FXR KO mice. (A) Principal component analyses of serum metabolomes of WT and FXR KO mice fed with either a CD or WD. (B) Venn diagrams show the numbers of distinct and overlapping metabolites that were changed by differential diets intake in 3 age groups (raw p value < 0.05 and FDR < 0.1). The metabolites in purple were affected by diet in both genotypes. Fig. S6. The effects of diets on urine metabolomes in WT and FXR KO mice. (A) Principal component analyses of urine metabolomes of WT and FXR KO mice. (B) Venn diagrams show the numbers of distinct and overlapping metabolites that were changed due to differential diet intake in 3 age groups (raw p value < 0.05 and FDR < 0.1). The metabolites in purple are commonly affected by diet in both genotypes. Fig. S7. The effects of diets on cecal microbiota. (A) Principal component analyses of cecal microbiota at genus level of WT and FXR KO mice fed with either a CD or WD. (B) Venn diagrams show the numbers of distinct and overlapping cecal microbiota at the genus level that were changed by differential diets intake in 3 age groups (raw p value < 0.05). Fig. S8. [file 40364_2023_458_MOESM2_ESM.pdf]

Fig.S2

(A)

WT
WD *vs.* CD (36)

5	10	15	Gene
7.81	2.21	2.78	*Adam11*
5.31	2.72	4.73	*Cyp3a11*
4.01	6.44	4.87	*Gramd1b*
3.64	2.49	2.03	*Cyp3a59*
2.95	2.12	2.72	*Orm3*
2.79	2.07	2.32	*Pde5a*
2.63	2.33	5.64	*Slc39a4*
2.21	3.84	5.96	*Adgrv1*

Fold change

5	10	15	Gene
0.02	0.14	0.11	*Sqle*
0.05	0.05	0.06	*Glra3*
0.10	0.25	0.12	*Srgap3*
0.11	0.32	0.24	*D830032E09Rik*
0.11	0.35	0.22	*Mvd*
0.14	0.40	0.29	*Ccdc69*
0.15	0.48	0.36	*Fdps*
0.15	0.22	0.24	*Pdzk1ip1*
0.16	0.41	0.29	*Nsdhl*
0.17	0.10	0.03	*Nat8f7*
0.18	0.41	0.09	*Acpp*
0.18	0.47	0.21	*Rdh11*
0.20	0.35	0.22	*Gm32468*
0.21	0.41	0.17	*Gm49012*
0.23	0.44	0.34	*Msmo1*
0.30	0.39	0.45	*Lmntd2*
0.32	0.13	0.25	*Hamp2*
0.33	0.35	0.39	*Tspyl4*
0.33	0.04	0.16	*Cyp2c69*
0.35	0.18	0.17	*Cyp2c40*
0.35	0.37	0.25	*Fras1*
0.36	0.47	0.40	*Fdft1*
0.37	0.38	0.34	*Tm7sf2*
0.39	0.45	0.28	*Gm10642*
0.41	0.34	0.47	*Fasn*
0.49	0.42	0.28	*Nat8f6*
0.50	0.30	0.43	*Rec8*

(B)

FXR KO
WD *vs.* CD (6)

5	10	15	Gene
19.56	4.14	61.39	*Scd3*
6.63	4.17	2.81	*Cidec*
3.63	2.06	2.13	*9130409I23Rik*
2.93	2.33	2.87	*Csad*
0.41	0.47	0.30	*Cyp39a1*
0.37	0.45	0.46	*Dntt*

Fold change

Fig. S3

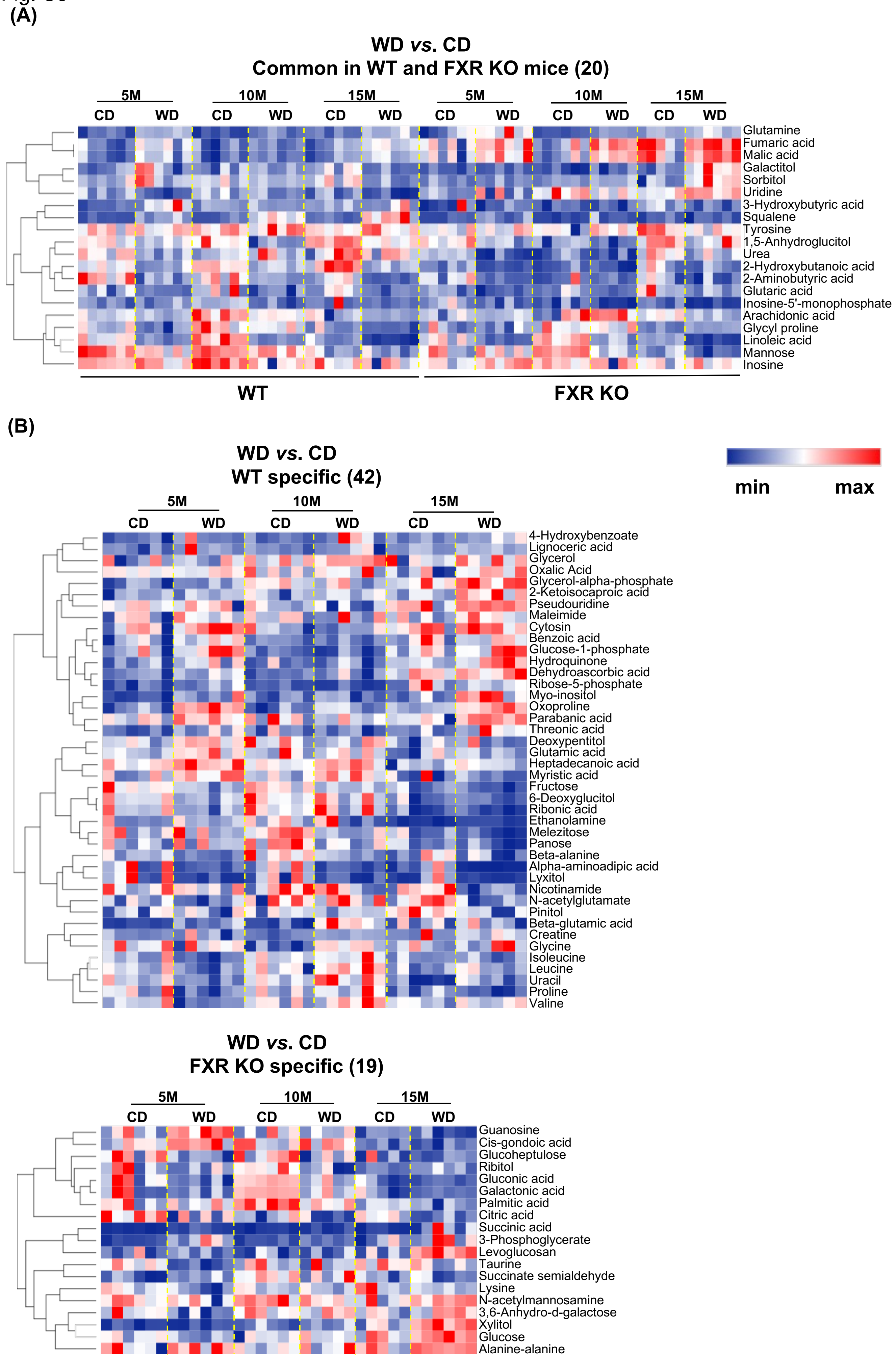
(A)
WD vs. CD
Common in WT and FXR KO mice (20)
5M
10M
15M
5M
10M
15M
CD
WD
CD
WD
CD
WD
CD
WD
CD
WD
CD
WD
Glutamine
Fumaric acid
Malic acid
Galactitol
Sorbitol
Uridine
3-Hydroxybutyric acid
Squalene
Tyrosine
1,5-Anhydroglucitol
Urea
2-Hydroxybutanoic acid
2-Aminobutyric acid
Glutaric acid
Inosine-5'-monophosphate
Arachidonic acid
Glycyl proline
Linoleic acid
Mannose
Inosine
WT
FXR KO
(B)
WD vs. CD
WT specific (42)
min
max
5M
10M
15M
CD
WD
CD
WD
CD
WD
4-Hydroxybenzoate
Lignoceric acid
Glycerol
Oxalic Acid
Glycerol-alpha-phosphate
2-Ketoisocaproic acid
Pseudouridine
Maleimide
Cytosin
Benzoic acid
Glucose-1-phosphate
Hydroquinone
Dehydroascorbic acid
Ribose-5-phosphate
Myo-inositol
Oxoproline
Parabanic acid
Threonic acid
Deoxypentitol
Glutamic acid
Heptadecanoic acid
Myristic acid
Fructose
6-Deoxyglucitol
Ribonic acid
Ethanolamine
Melezitose
Panose
Beta-alanine
Alpha-aminoadipic acid
Lyxitol
Nicotinamide
N-acetylglutamate
Pinitol
Beta-glutamic acid
Creatine
Glycine
Isoleucine
Leucine
Uracil
Proline
Valine
WD vs. CD
FXR KO specific (19)
5M
10M
15M
CD
WD
CD
WD
CD
WD
Guanosine
Cis-gondoic acid
Glucoheptulose
Ribitol
Gluconic acid
Galactonic acid
Palmitic acid
Citric acid
Succinic acid
3-Phosphoglycerate
Levoglucosan
Taurine
Succinate semialdehyde
Lysine
N-acetylmannosamine
3,6-Anhydro-d-galactose
Xylitol
Glucose
Alanine-alanine

Fig. S4

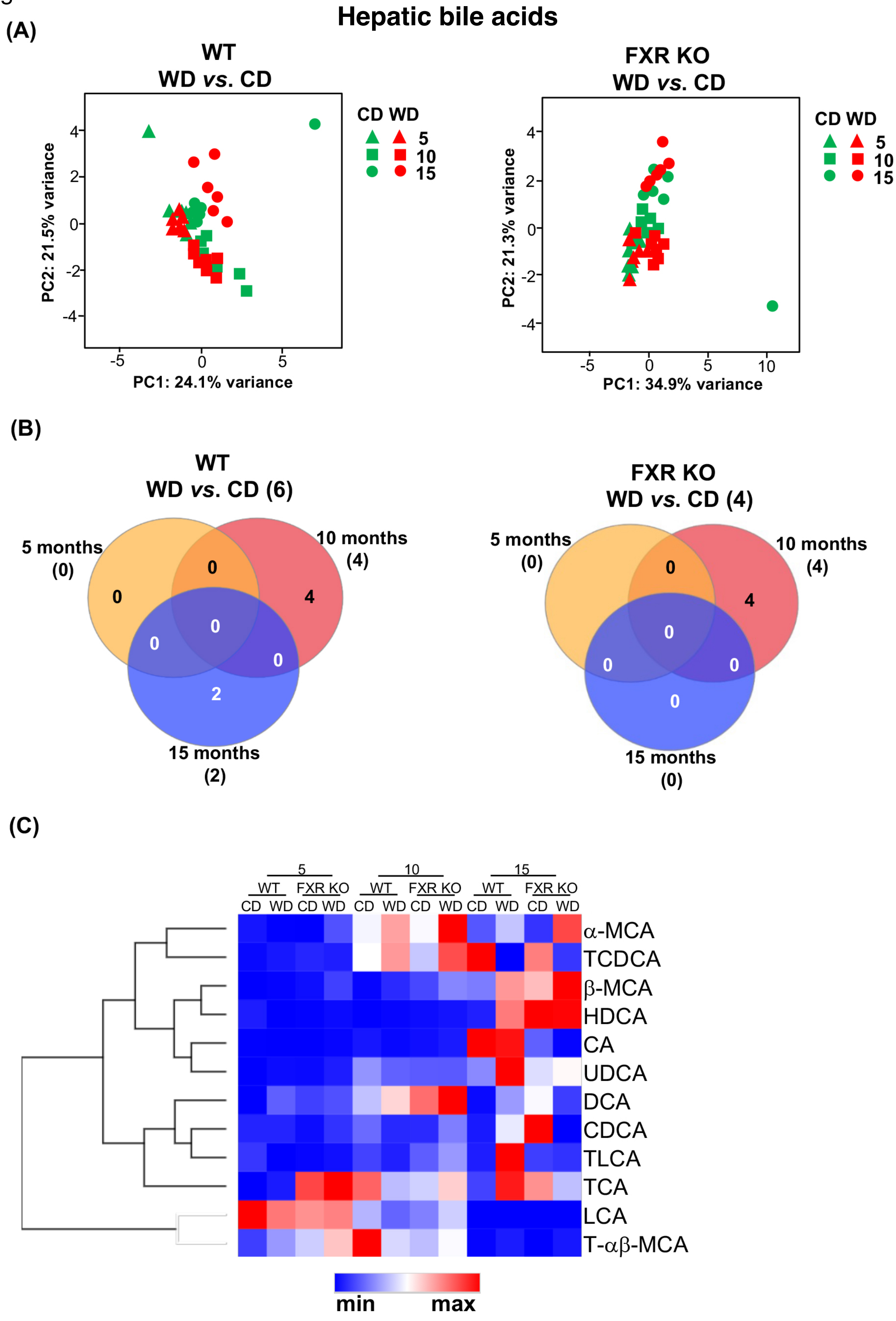
Hepatic bile acids
(A)
WT
WD vs. CD
PC2: 21.5% variance
PC1: 24.1% variance
CD WD
5
10
15
FXR KO
WD vs. CD
PC2: 21.3% variance
PC1: 34.9% variance
(B)
WT
WD vs. CD (6)
5 months (0)
10 months (4)
15 months (2)
FXR KO
WD vs. CD (4)
5 months (0)
10 months (4)
15 months (0)
(C)
5
10
15
WT
FXR KO
CD WD CD WD
α-MCA
TCDCA
β-MCA
HDCA
CA
UDCA
DCA
CDCA
TLCA
TCA
LCA
T-αβ-MCA
min
max

Fig. S5

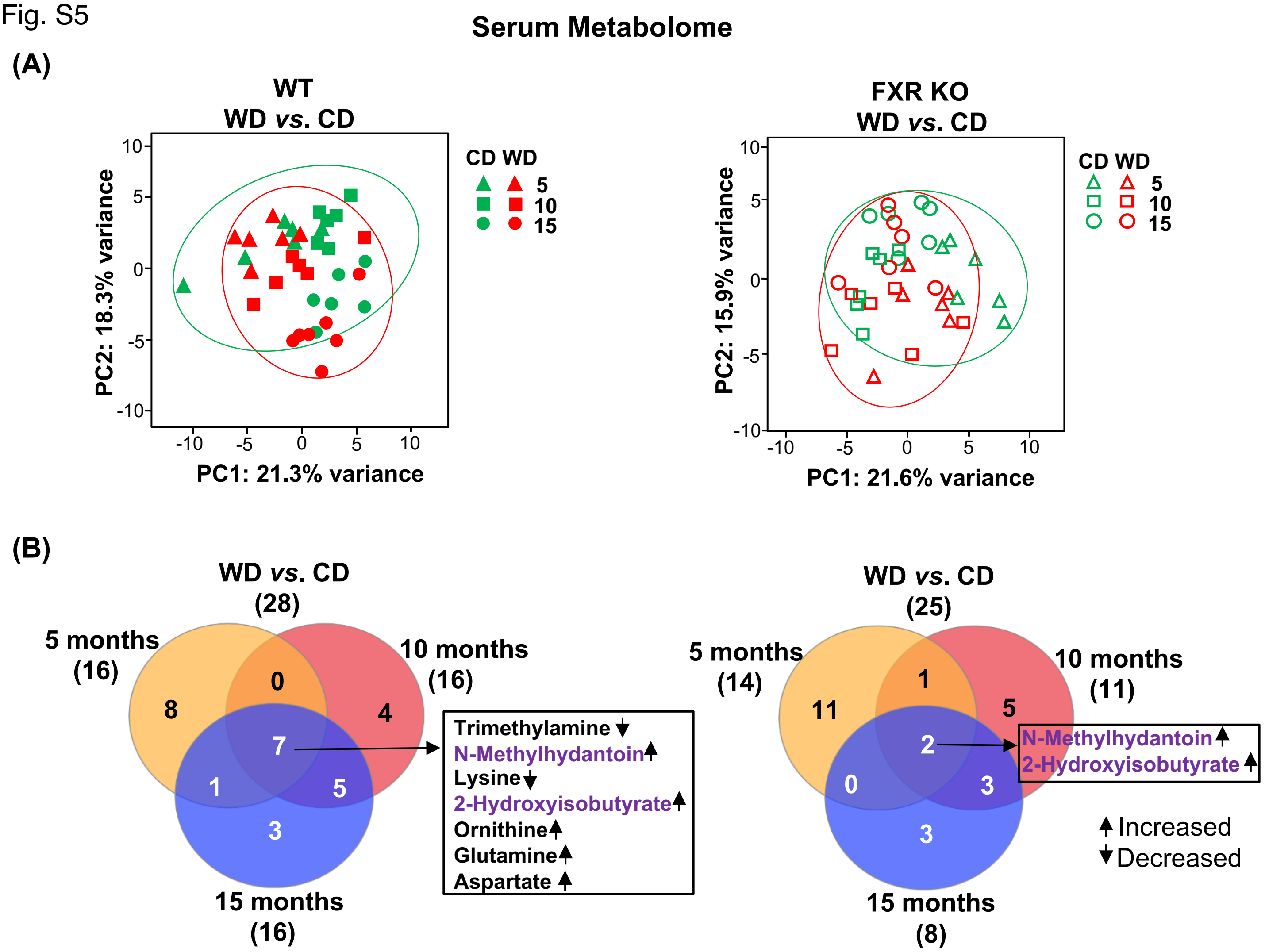

Serum Metabolome
(A)
WT
WD vs. CD
PC1: 21.3% variance
PC2: 18.3% variance
CD WD
5
10
15
FXR KO
WD vs. CD
PC1: 21.6% variance
PC2: 15.9% variance
CD WD
5
10
15
(B)
WD vs. CD
(28)
5 months
(16)
10 months
(16)
15 months
(16)
8
0
4
7
1
5
3
Trimethylamine ↓
N-Methylhydantoin ↑
Lysine ↓
2-Hydroxyisobutyrate ↑
Ornithine ↑
Glutamine ↑
Aspartate ↑
WD vs. CD
(25)
5 months
(14)
10 months
(11)
15 months
(8)
11
1
5
2
0
3
3
N-Methylhydantoin ↑
2-Hydroxyisobutyrate ↑
↑ Increased
↓ Decreased

Fig. S6

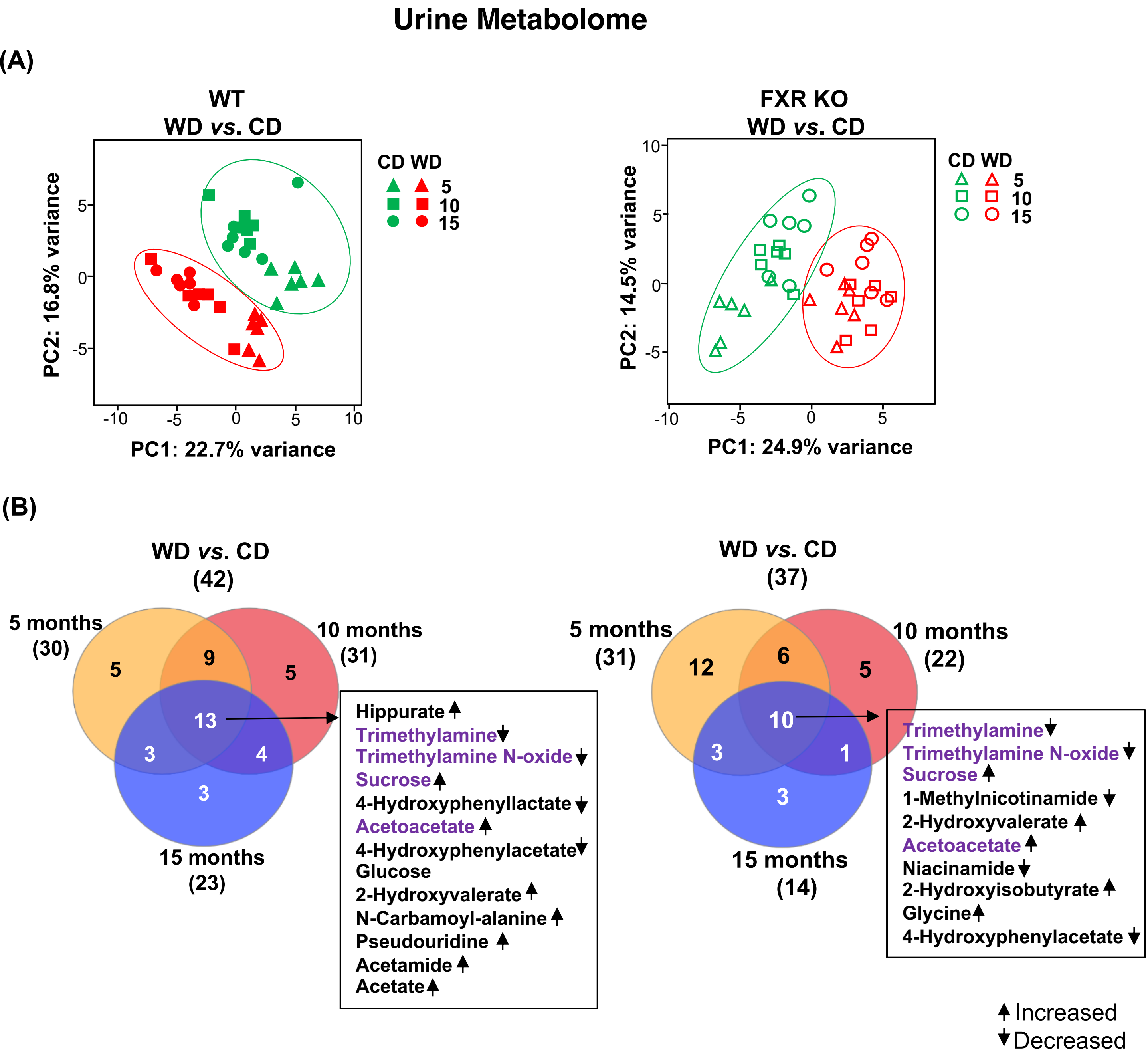

Urine Metabolome
(A)
WT
WD vs. CD
PC2: 16.8% variance
PC1: 22.7% variance
CD WD
5
10
15
FXR KO
WD vs. CD
PC2: 14.5% variance
PC1: 24.9% variance
CD WD
5
10
15
(B)
WD vs. CD
(42)
5 months
(30)
10 months
(31)
15 months
(23)
5
9
5
13
3
4
3
Hippurate ↑
Trimethylamine ↓
Trimethylamine N-oxide ↓
Sucrose ↑
4-Hydroxyphenyllactate ↓
Acetoacetate ↑
4-Hydroxyphenylacetate ↓
Glucose
2-Hydroxyvalerate ↑
N-Carbamoyl-alanine ↑
Pseudouridine ↑
Acetamide ↑
Acetate ↑
WD vs. CD
(37)
5 months
(31)
10 months
(22)
15 months
(14)
12
6
5
10
3
1
3
Trimethylamine ↓
Trimethylamine N-oxide ↓
Sucrose ↑
1-Methylnicotinamide ↓
2-Hydroxyvalerate ↑
Acetoacetate ↑
Niacinamide ↓
2-Hydroxyisobutyrate ↑
Glycine ↑
4-Hydroxyphenylacetate ↓
↑ Increased
↓ Decreased

Fig. S7

Cecal microbiota at genus level

(A)

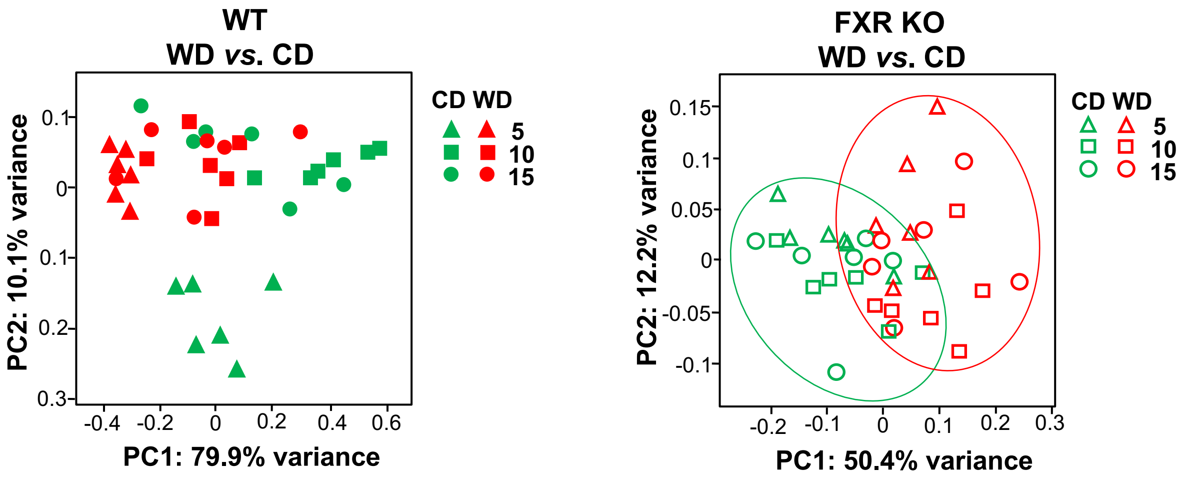

(B)

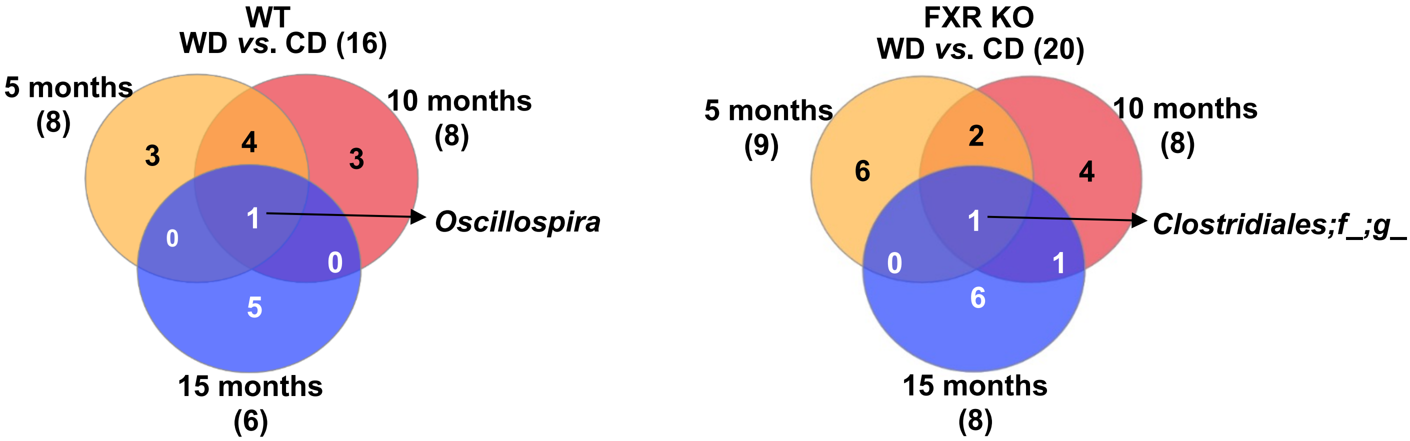

Fig. S8

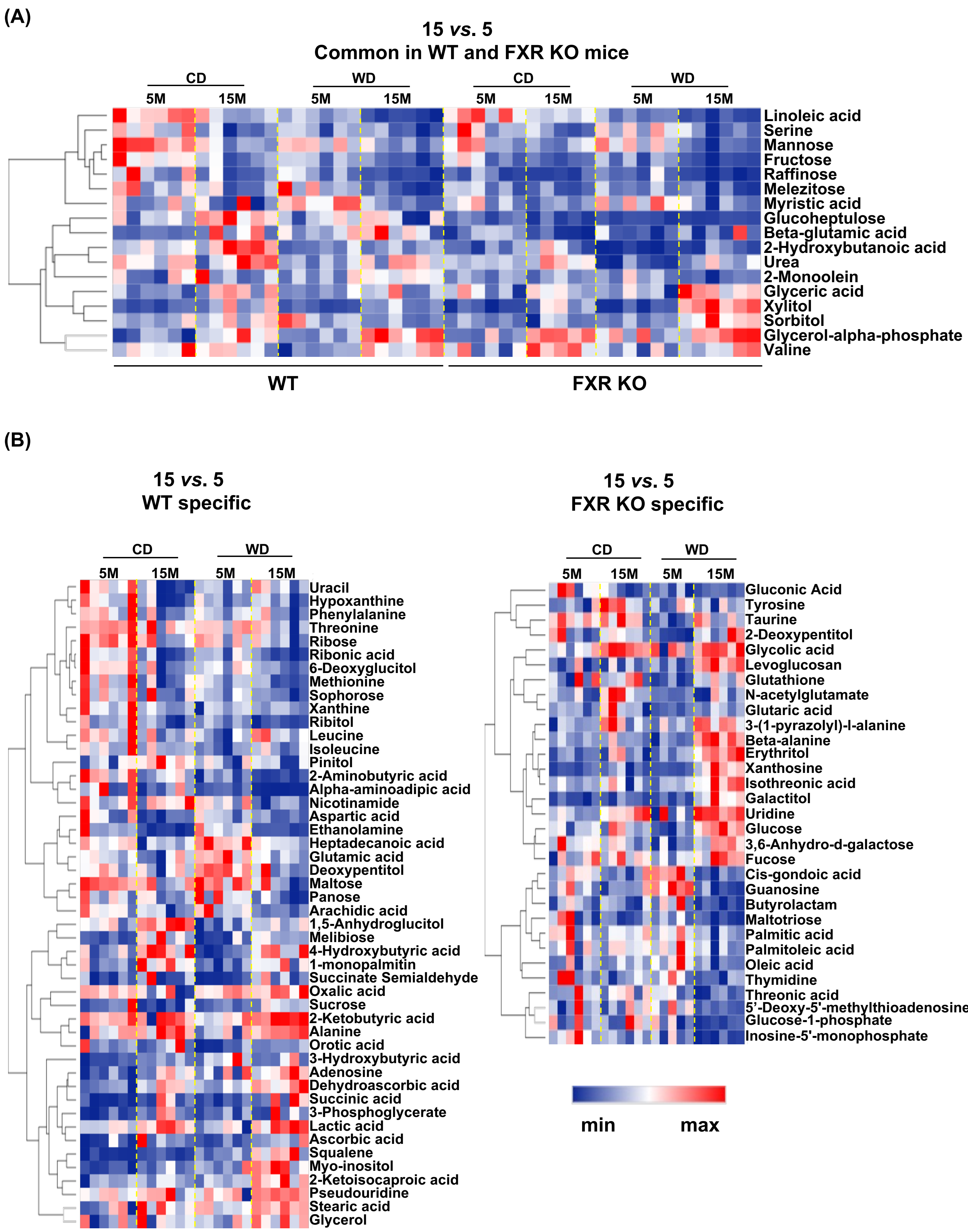
(A)
15 vs. 5
Common in WT and FXR KO mice
CD
WD
CD
WD
5M
15M
5M
15M
5M
15M
5M
15M
Linoleic acid
Serine
Mannose
Fructose
Raffinose
Melezitose
Myristic acid
Glucoheptulose
Beta-glutamic acid
2-Hydroxybutanoic acid
Urea
2-Monoolein
Glyceric acid
Xylitol
Sorbitol
Glycerol-alpha-phosphate
Valine
WT
FXR KO
(B)
15 vs. 5
WT specific
CD
WD
5M
15M
5M
15M
Uracil
Hypoxanthine
Phenylalanine
Threonine
Ribose
Ribonic acid
6-Deoxyglucitol
Methionine
Sophorose
Xanthine
Ribitol
Leucine
Isoleucine
Pinitol
2-Aminobutyric acid
Alpha-aminoadipic acid
Nicotinamide
Aspartic acid
Ethanolamine
Heptadecanoic acid
Glutamic acid
Deoxypentitol
Maltose
Panose
Arachidic acid
1,5-Anhydroglucitol
Melibiose
4-Hydroxybutyric acid
1-monopalmitin
Succinate Semialdehyde
Oxalic acid
Sucrose
2-Ketobutyric acid
Alanine
Orotic acid
3-Hydroxybutyric acid
Adenosine
Dehydroascorbic acid
Succinic acid
3-Phosphoglycerate
Lactic acid
Ascorbic acid
Squalene
Myo-inositol
2-Ketoisocaproic acid
Pseudouridine
Stearic acid
Glycerol
15 vs. 5
FXR KO specific
CD
WD
5M
15M
5M
15M
Gluconic Acid
Tyrosine
Taurine
2-Deoxypentitol
Glycolic acid
Levoglucosan
Glutathione
N-acetylglutamate
Glutaric acid
3-(1-pyrazolyl)-l-alanine
Beta-alanine
Erythritol
Xanthosine
Isothreonic acid
Galactitol
Uridine
Glucose
3,6-Anhydro-d-galactose
Fucose
Cis-gondoic acid
Guanosine
Butyrolactam
Maltotriose
Palmitic acid
Palmitoleic acid
Oleic acid
Thymidine
Threonic acid
5'-Deoxy-5'-methylthioadenosine
Glucose-1-phosphate
Inosine-5'-monophosphate
min
max

Fig. S9

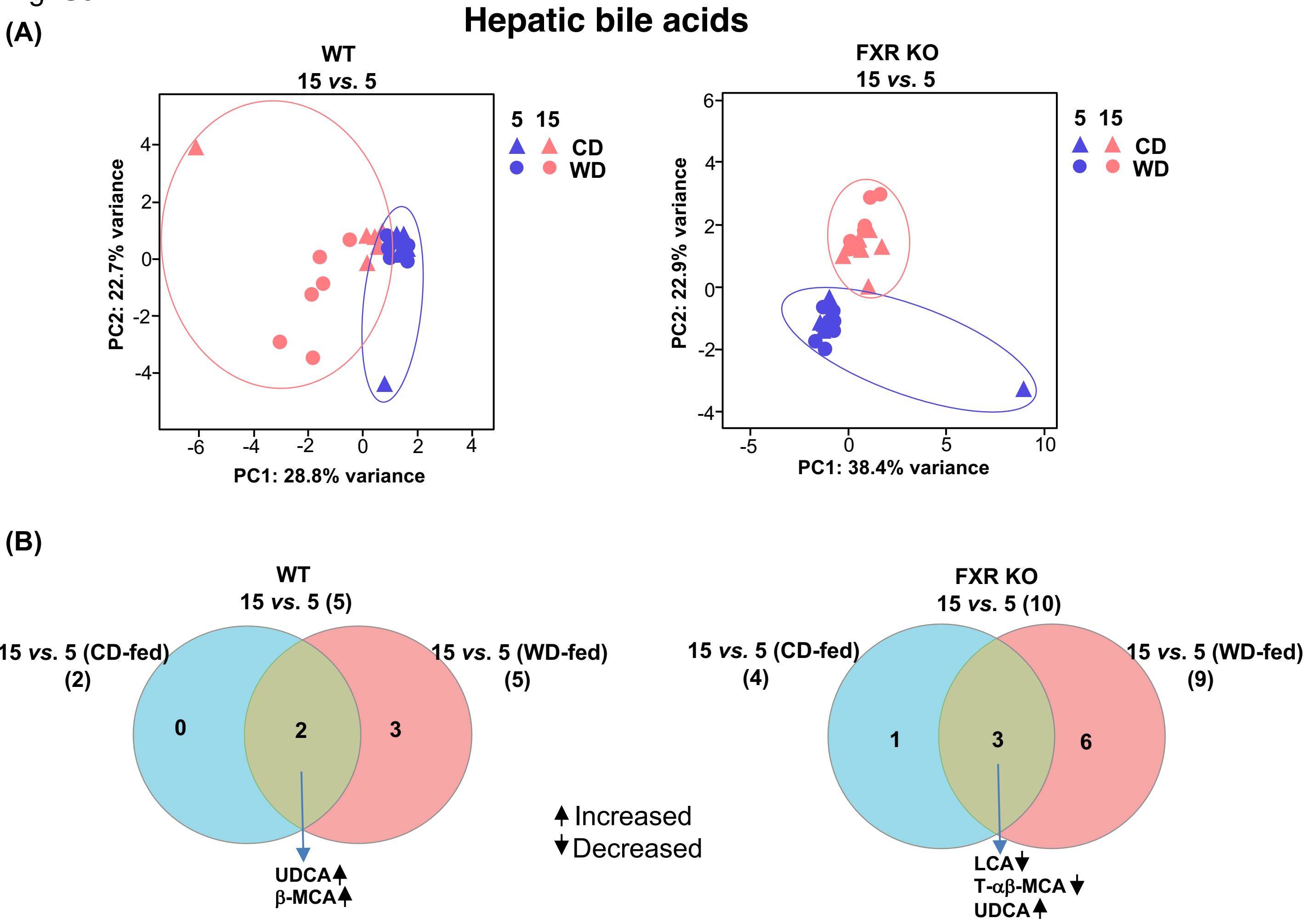

Hepatic bile acids
(A)
WT
15 vs. 5
PC1: 28.8% variance
PC2: 22.7% variance
5 15
CD
WD
FXR KO
15 vs. 5
PC1: 38.4% variance
PC2: 22.9% variance
5 15
CD
WD
(B)
WT
15 vs. 5 (5)
15 vs. 5 (CD-fed)
(2)
15 vs. 5 (WD-fed)
(5)
0
2
3
UDCA↑
β-MCA↑
↑Increased
↓Decreased
FXR KO
15 vs. 5 (10)
15 vs. 5 (CD-fed)
(4)
15 vs. 5 (WD-fed)
(9)
1
3
6
LCA↓
T-αβ-MCA↓
UDCA↑

Fig. S10

Serum Metabolome

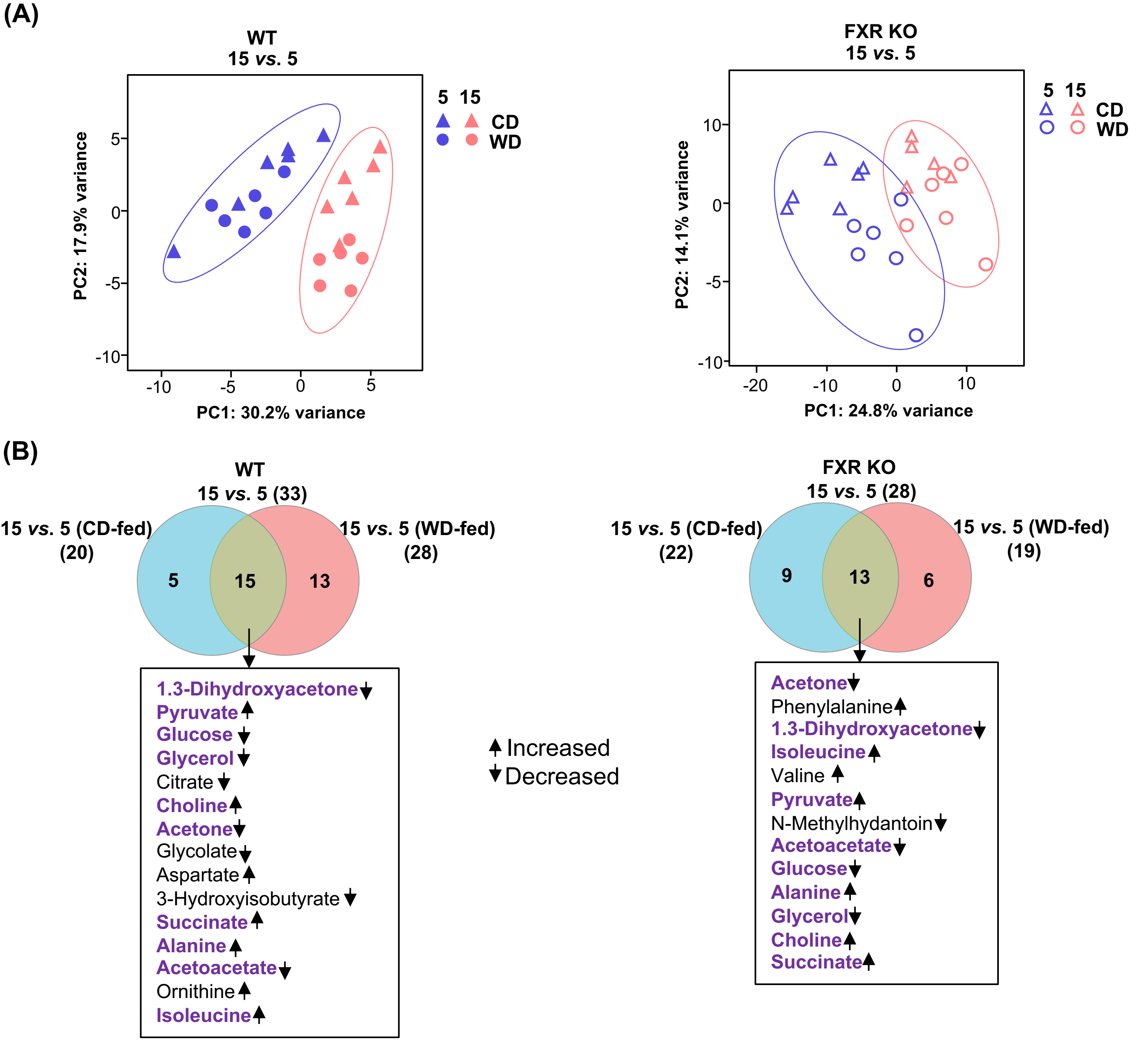

Fig. S11

Urine Metabolome

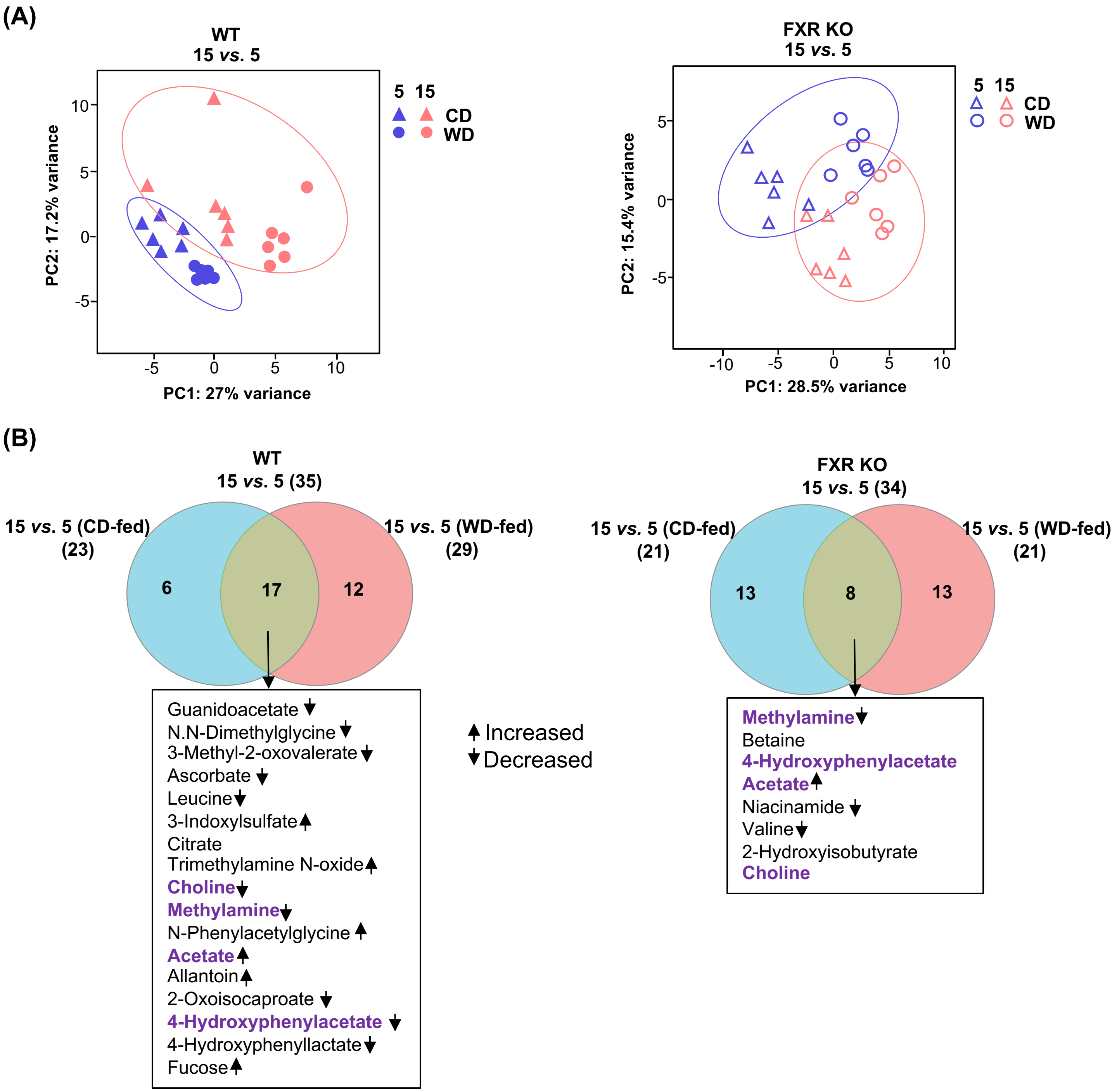

Fig. S12

Cecal microbiota at genus level

(A)

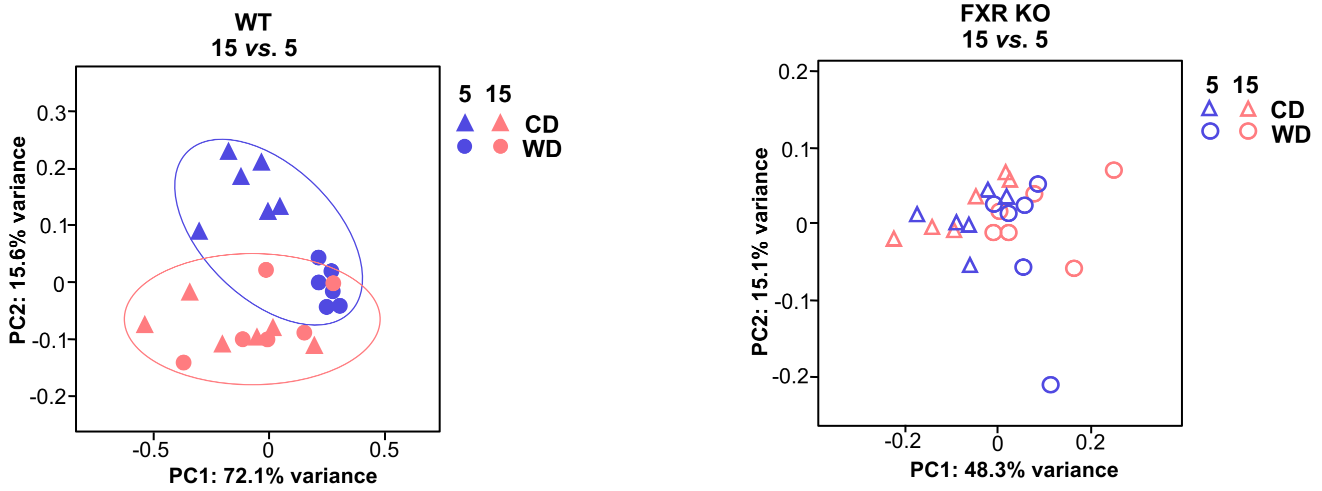

(B)

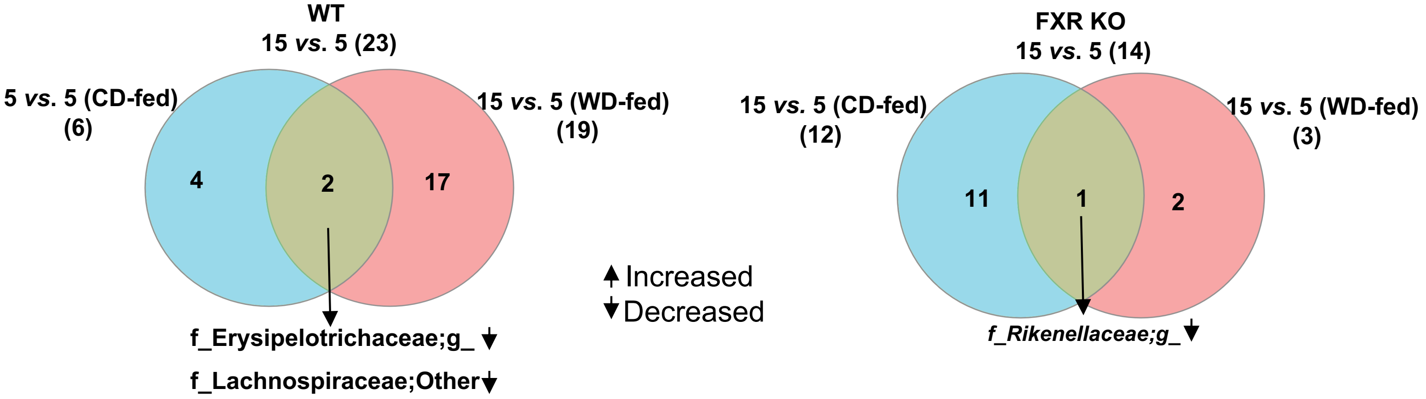

(A) Fig. S13

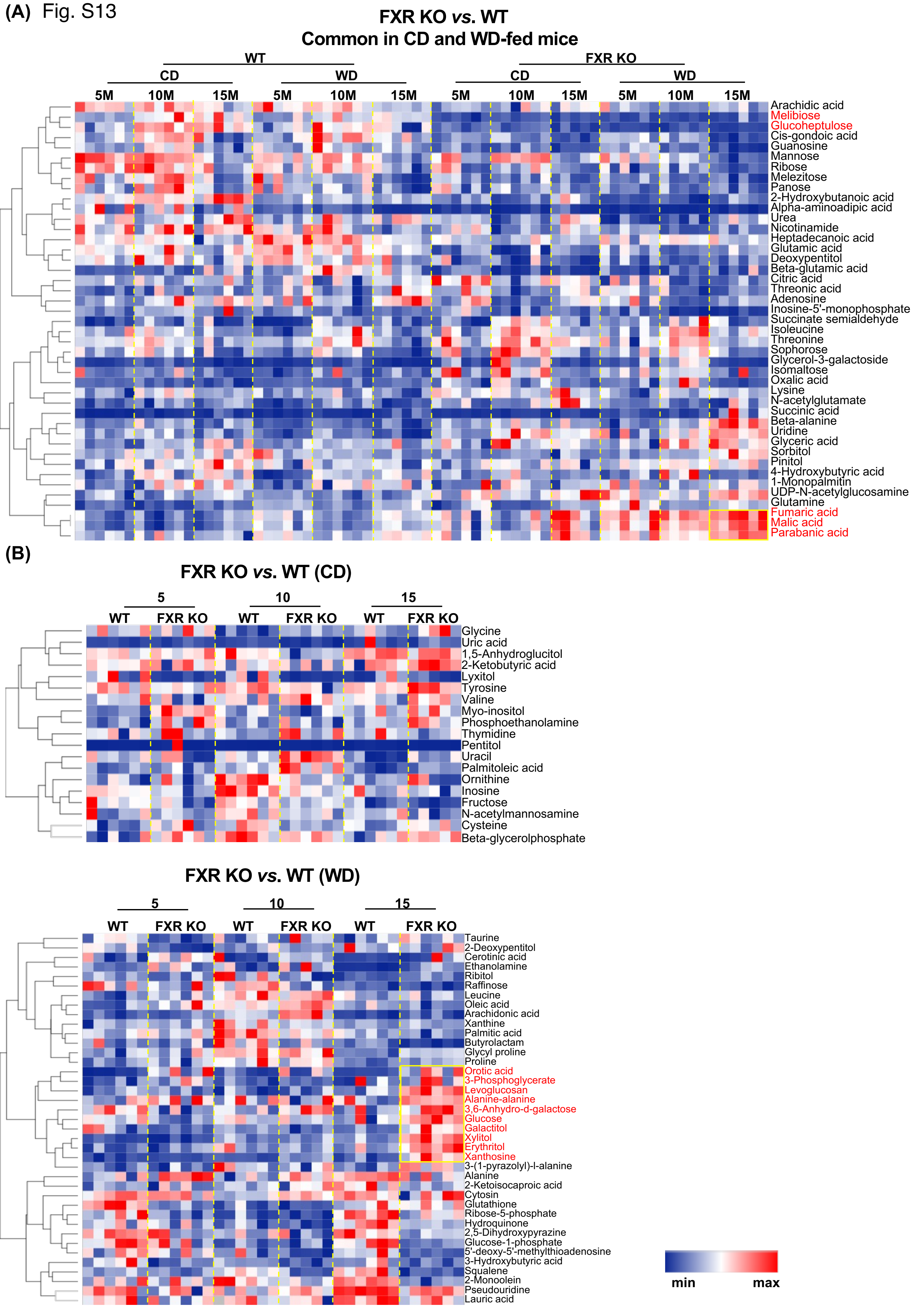
FXR KO vs. WT
Common in CD and WD-fed mice
WT
FXR KO
CD
WD
CD
WD
5M
10M
15M
5M
10M
15M
5M
10M
15M
5M
10M
15M
Arachidic acid
Melibiose
Glucoheptulose
Cis-gondoic acid
Guanosine
Mannose
Ribose
Melezitose
Panose
2-Hydroxybutanoic acid
Alpha-aminoadipic acid
Urea
Nicotinamide
Heptadecanoic acid
Glutamic acid
Deoxypentitol
Beta-glutamic acid
Citric acid
Threonic acid
Adenosine
Inosine-5'-monophosphate
Succinate semialdehyde
Isoleucine
Threonine
Sophorose
Glycerol-3-galactoside
Isomaltose
Oxalic acid
Lysine
N-acetylglutamate
Succinic acid
Beta-alanine
Uridine
Glyceric acid
Sorbitol
Pinitol
4-Hydroxybutyric acid
1-Monopalmitin
UDP-N-acetylglucosamine
Glutamine
Fumaric acid
Malic acid
Parabanic acid
(B)
FXR KO vs. WT (CD)
5
10
15
WT
FXR KO
WT
FXR KO
WT
FXR KO
Glycine
Uric acid
1,5-Anhydroglucitol
2-Ketobutyric acid
Lyxitol
Tyrosine
Valine
Myo-inositol
Phosphoethanolamine
Thymidine
Pentitol
Uracil
Palmitoleic acid
Ornithine
Inosine
Fructose
N-acetylmannosamine
Cysteine
Beta-glycerolphosphate
FXR KO vs. WT (WD)
5
10
15
WT
FXR KO
WT
FXR KO
WT
FXR KO
Taurine
2-Deoxypentitol
Cerotinic acid
Ethanolamine
Ribitol
Raffinose
Leucine
Oleic acid
Arachidonic acid
Xanthine
Palmitic acid
Butyrolactam
Glycyl proline
Proline
Orotic acid
3-Phosphoglycerate
Levoglucosan
Alanine-alanine
3,6-Anhydro-d-galactose
Glucose
Galactitol
Xylitol
Erythritol
Xanthosine
3-(1-pyrazolyl)-l-alanine
Alanine
2-Ketoisocaproic acid
Cytosin
Glutathione
Ribose-5-phosphate
Hydroquinone
2,5-Dihydroxypyrazine
Glucose-1-phosphate
5'-deoxy-5'-methylthioadenosine
3-Hydroxybutyric acid
Squalene
2-Monoolein
Pseudouridine
Lauric acid
min
max

Fig. S14

Hepatic bile acids

(A)

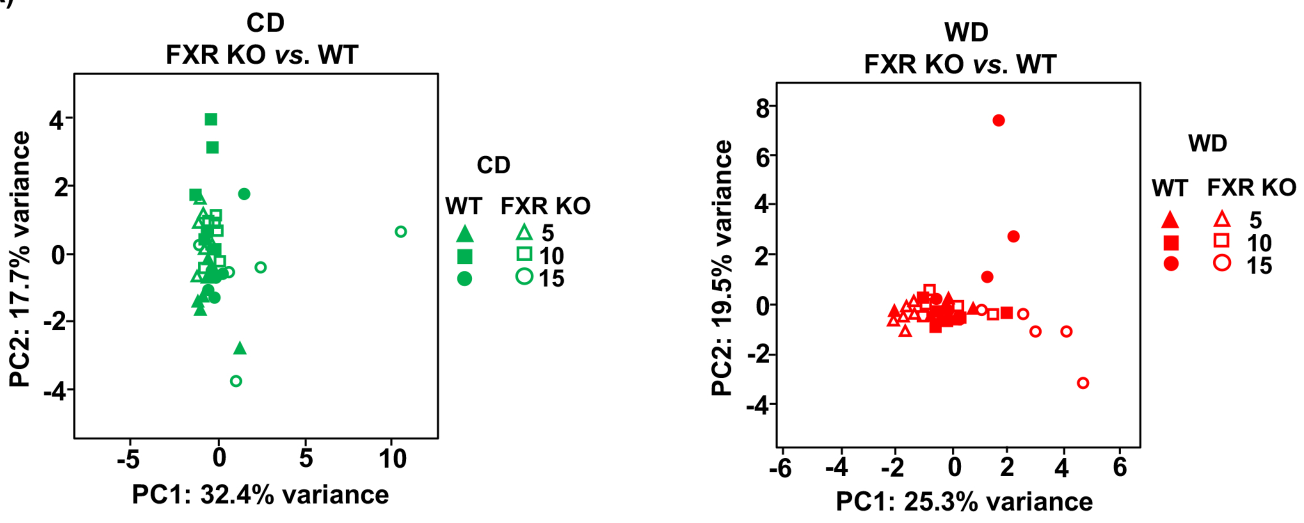

(B)

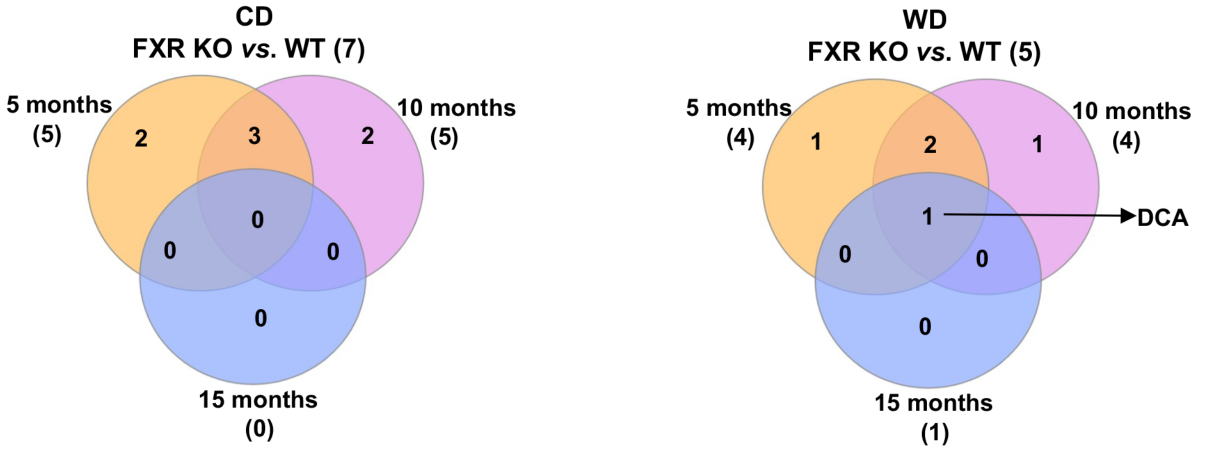

Fig. S15

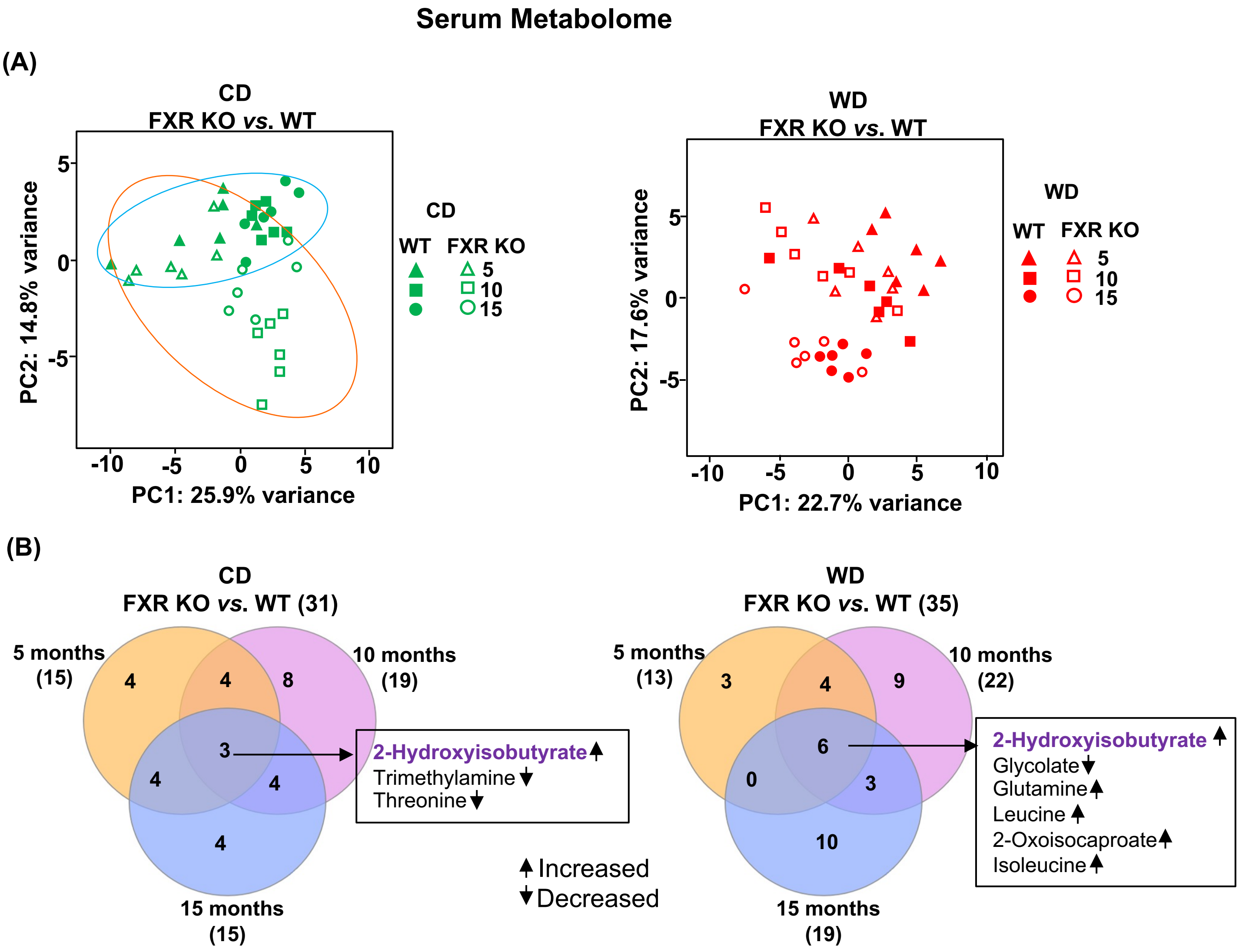
Serum Metabolome
(A)
CD
FXR KO vs. WT
PC2: 14.8% variance
PC1: 25.9% variance
CD
WT
FXR KO
5
10
15
WD
FXR KO vs. WT
PC2: 17.6% variance
PC1: 22.7% variance
WD
WT
FXR KO
5
10
15
(B)
CD
FXR KO vs. WT (31)
5 months (15)
10 months (19)
15 months (15)
2-Hydroxyisobutyrate ↑
Trimethylamine ↓
Threonine ↓
↑ Increased
↓ Decreased
WD
FXR KO vs. WT (35)
5 months (13)
10 months (22)
15 months (19)
2-Hydroxyisobutyrate ↑
Glycolate ↓
Glutamine ↑
Leucine ↑
2-Oxoisocaproate ↑
Isoleucine ↑

Fig. S16

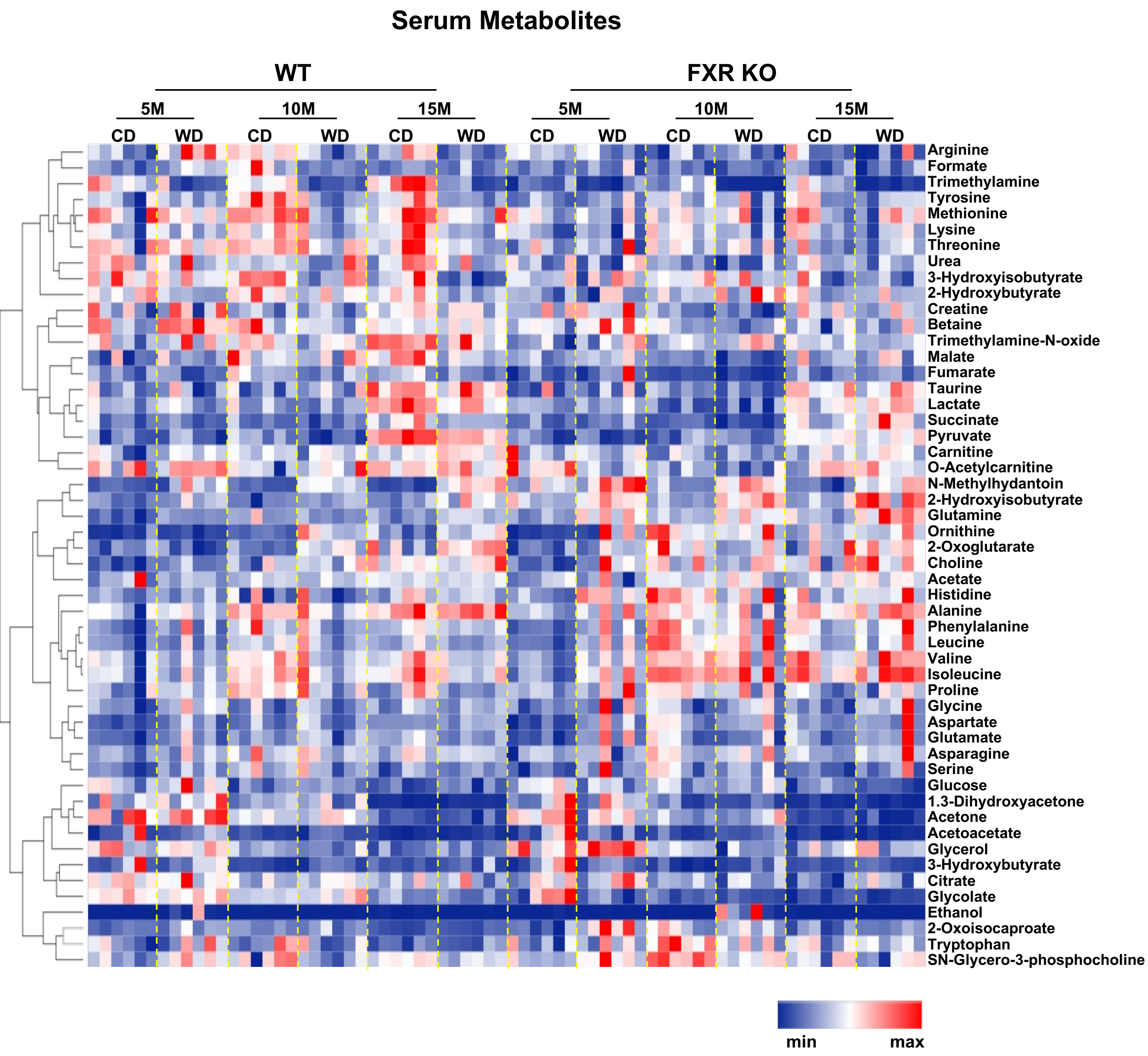
Serum Metabolites
WT
FXR KO
5M
10M
15M
5M
10M
15M
CD
WD
CD
WD
CD
WD
CD
WD
CD
WD
CD
WD
Arginine
Formate
Trimethylamine
Tyrosine
Methionine
Lysine
Threonine
Urea
3-Hydroxyisobutyrate
2-Hydroxybutyrate
Creatine
Betaine
Trimethylamine-N-oxide
Malate
Fumarate
Taurine
Lactate
Succinate
Pyruvate
Carnitine
O-Acetylcarnitine
N-Methylhydantoin
2-Hydroxyisobutyrate
Glutamine
Ornithine
2-Oxoglutarate
Choline
Acetate
Histidine
Alanine
Phenylalanine
Leucine
Valine
Isoleucine
Proline
Glycine
Aspartate
Glutamate
Asparagine
Serine
Glucose
1.3-Dihydroxyacetone
Acetone
Acetoacetate
Glycerol
3-Hydroxybutyrate
Citrate
Glycolate
Ethanol
2-Oxoisocaproate
Tryptophan
SN-Glycero-3-phosphocholine
min
max

Fig. S17

Urine Metabolome

(A)

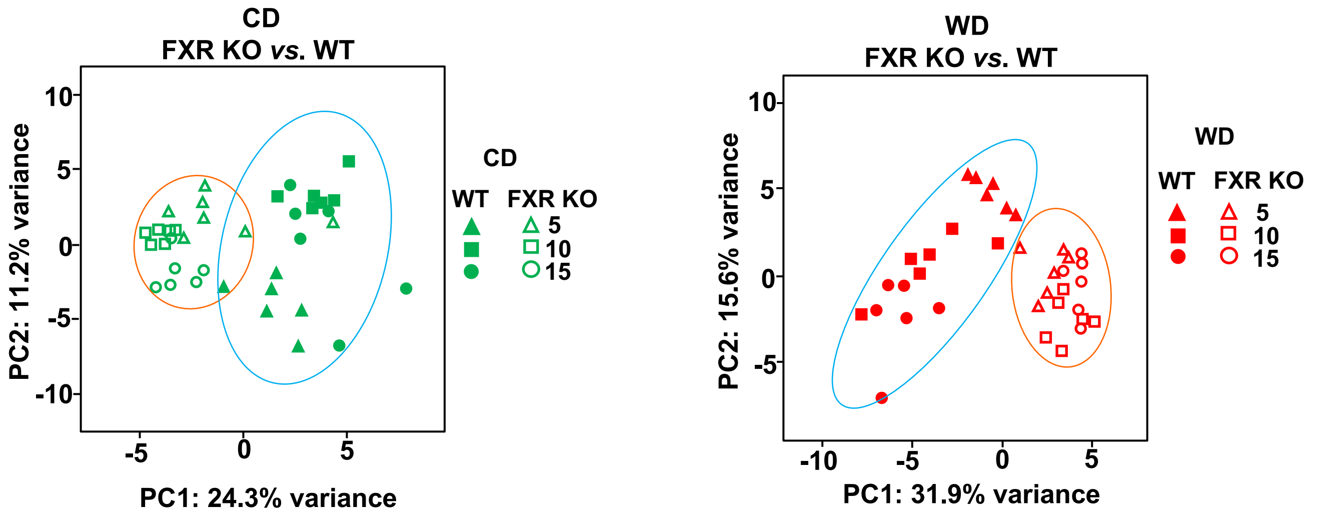

(B)

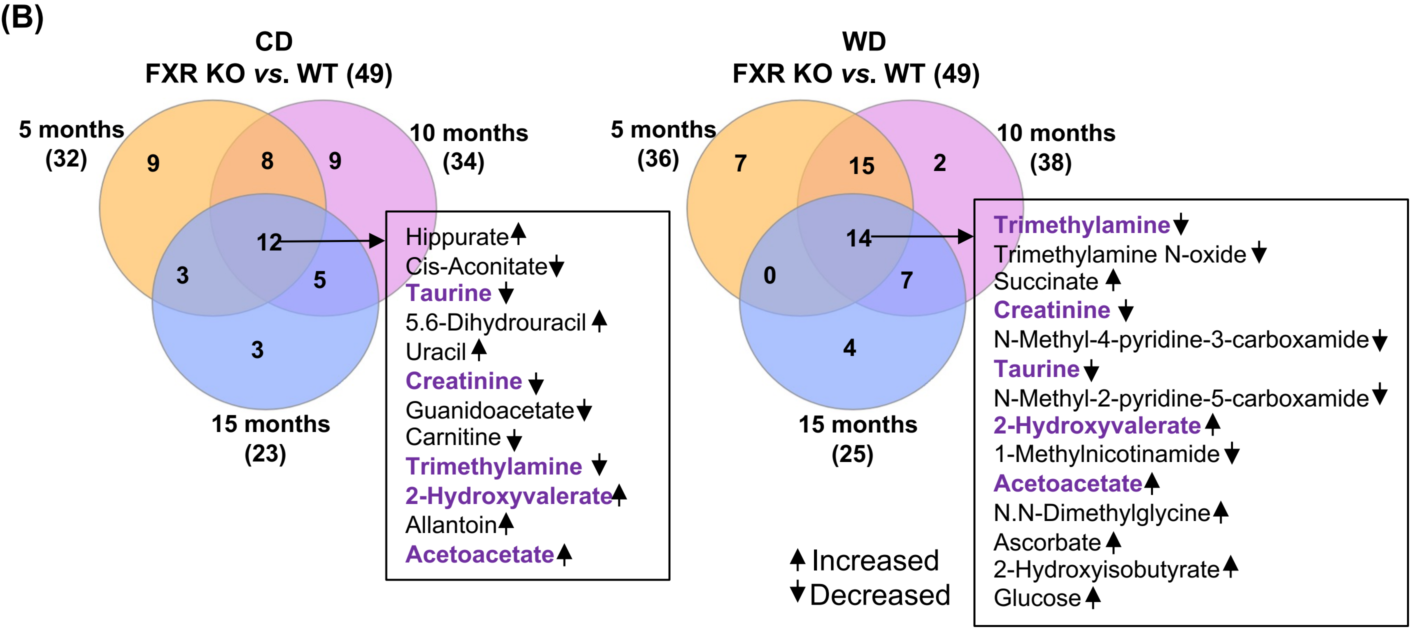

Fig. S18

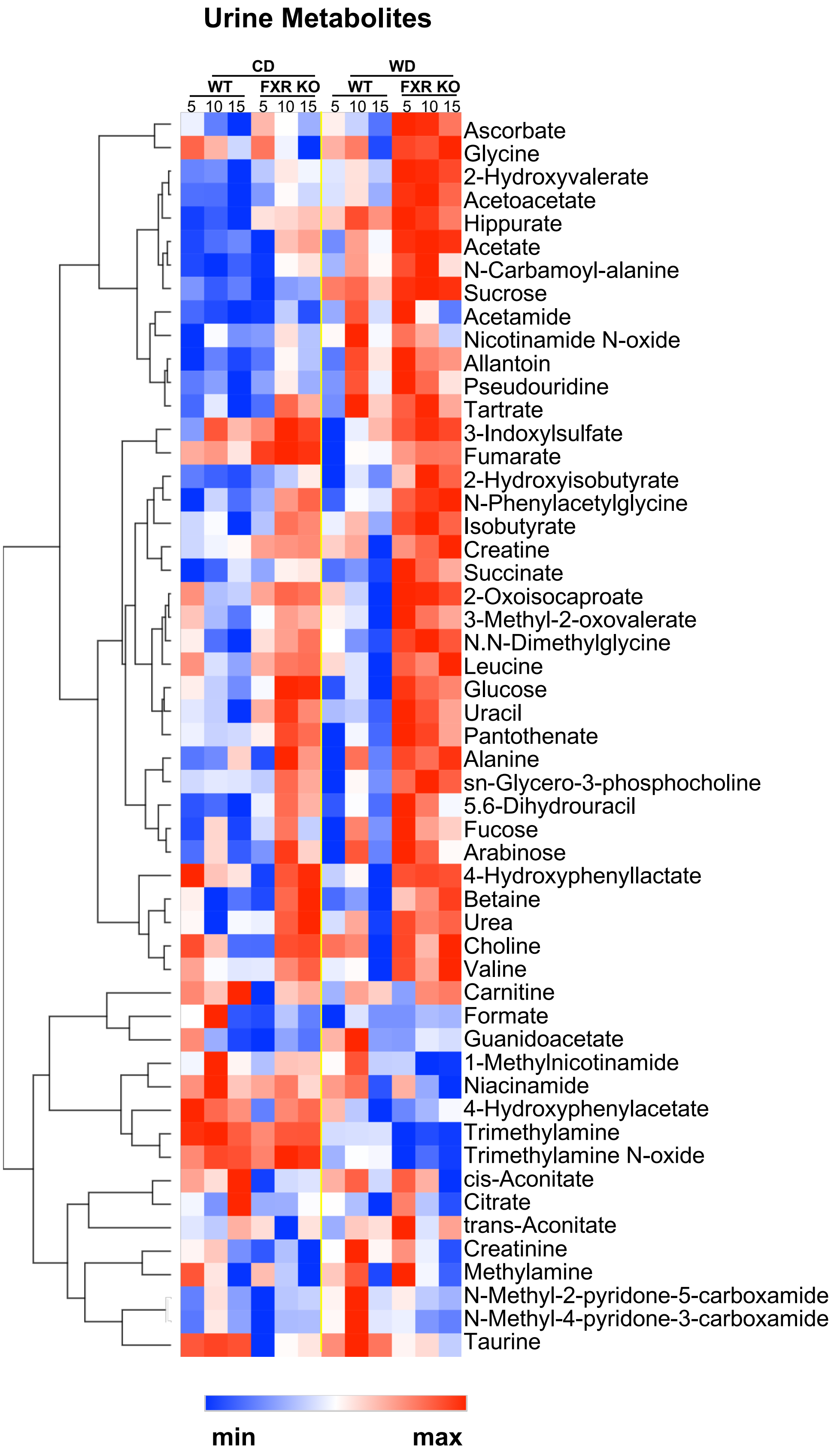
Urine Metabolites
CD
WD
WT
FXR KO
WT
FXR KO
5 10 15 5 10 15 5 10 15 5 10 15
Ascorbate
Glycine
2-Hydroxyvalerate
Acetoacetate
Hippurate
Acetate
N-Carbamoyl-alanine
Sucrose
Acetamide
Nicotinamide N-oxide
Allantoin
Pseudouridine
Tartrate
3-Indoxylsulfate
Fumarate
2-Hydroxyisobutyrate
N-Phenylacetylglycine
Isobutyrate
Creatine
Succinate
2-Oxoisocaproate
3-Methyl-2-oxovalerate
N.N-Dimethylglycine
Leucine
Glucose
Uracil
Pantothenate
Alanine
sn-Glycero-3-phosphocholine
5.6-Dihydrouracil
Fucose
Arabinose
4-Hydroxyphenyllactate
Betaine
Urea
Choline
Valine
Carnitine
Formate
Guanidoacetate
1-Methylnicotinamide
Niacinamide
4-Hydroxyphenylacetate
Trimethylamine
Trimethylamine N-oxide
cis-Aconitate
Citrate
trans-Aconitate
Creatinine
Methylamine
N-Methyl-2-pyridone-5-carboxamide
N-Methyl-4-pyridone-3-carboxamide
Taurine
min
max

Fig. S19

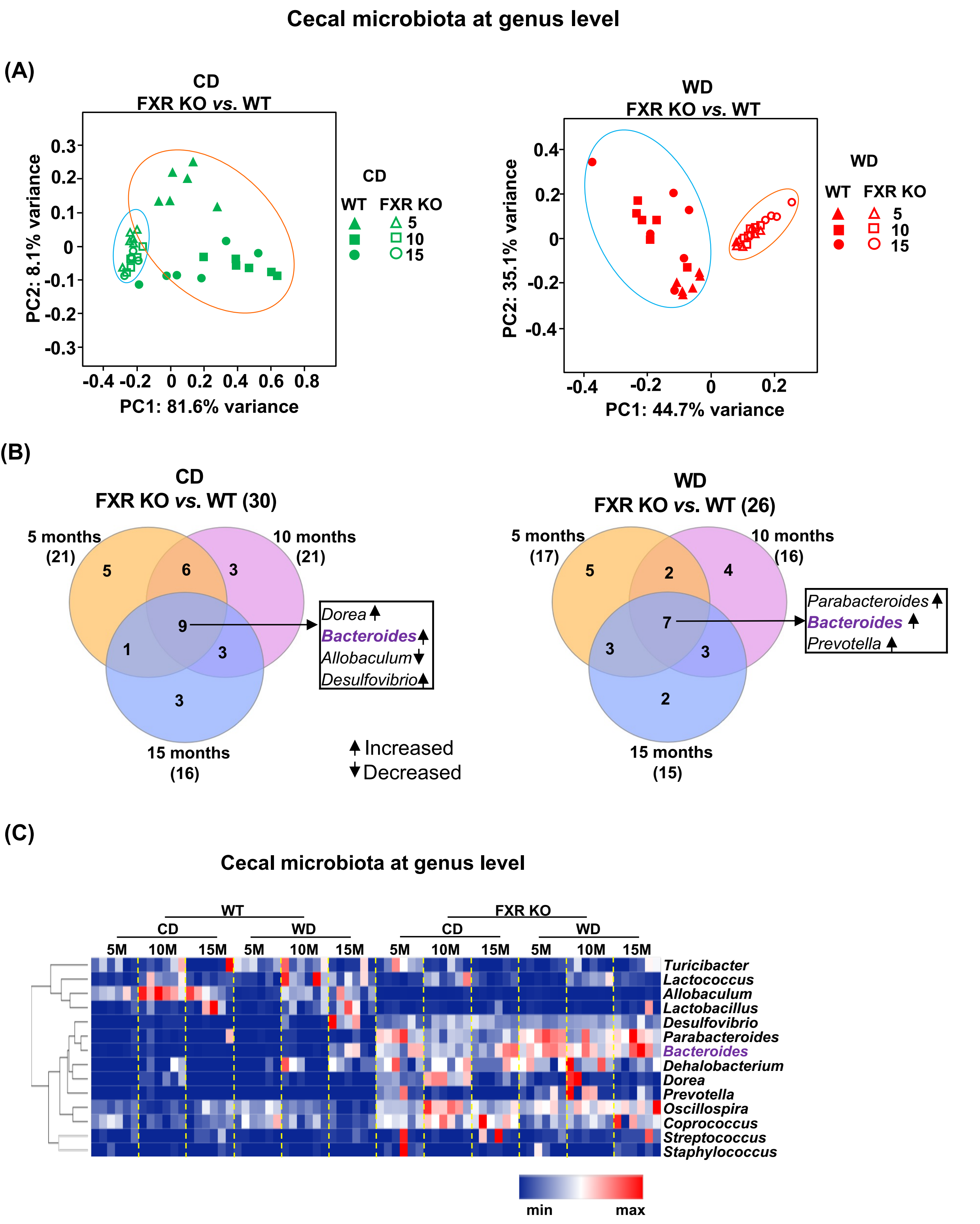
Cecal microbiota at genus level
(A)
CD
FXR KO vs. WT
PC2: 8.1% variance
PC1: 81.6% variance
0.3
0.2
0.1
0
-0.1
-0.2
-0.3
-0.4 -0.2 0 0.2 0.4 0.6 0.8
CD
WT FXR KO
5
10
15
WD
FXR KO vs. WT
PC2: 35.1% variance
PC1: 44.7% variance
0.4
0.2
0
-0.2
-0.4
-0.4 -0.2 0 0.2
WD
WT FXR KO
5
10
15
(B)
CD
FXR KO vs. WT (30)
5 months (21)
10 months (21)
15 months (16)
5
6
3
9
1
3
3
Dorea
Bacteroides
Allobaculum
Desulfovibrio
Increased
Decreased
WD
FXR KO vs. WT (26)
5 months (17)
10 months (16)
15 months (15)
5
2
4
7
3
3
2
Parabacteroides
Bacteroides
Prevotella
(C)
Cecal microbiota at genus level
WT
FXR KO
CD
WD
5M 10M 15M 5M 10M 15M 5M 10M 15M 5M 10M 15M
Turicibacter
Lactococcus
Allobaculum
Lactobacillus
Desulfovibrio
Parabacteroides
Bacteroides
Dehalobacterium
Dorea
Prevotella
Oscillospira
Coprococcus
Streptococcus
Staphylococcus
min
max

Fig. S20

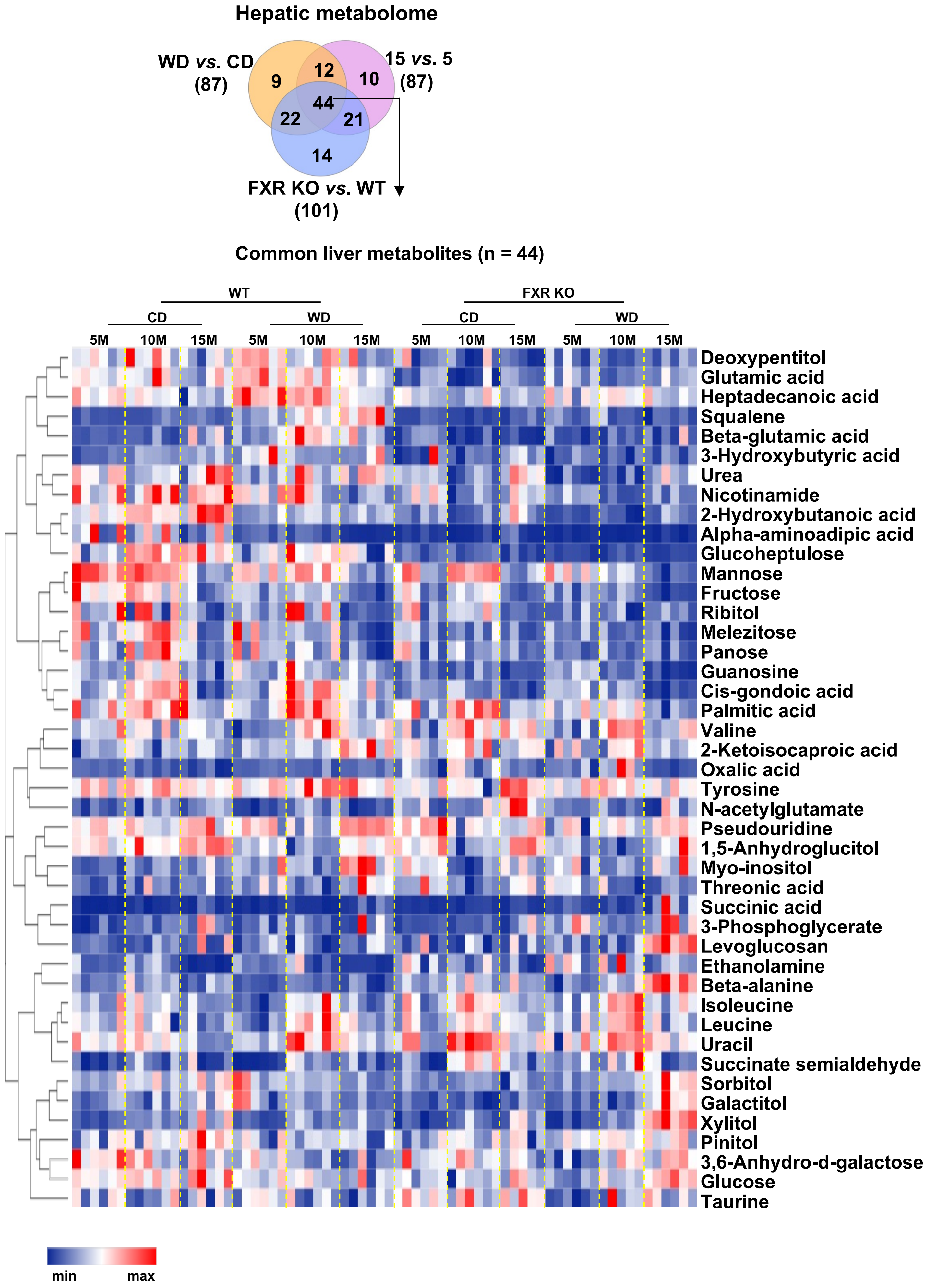

Hepatic metabolome
WD vs. CD
(87)
15 vs. 5
(87)
FXR KO vs. WT
(101)
9
12
10
44
22
21
14
Common liver metabolites (n = 44)
WT
FXR KO
CD
WD
CD
WD
5M
10M
15M
5M
10M
15M
5M
10M
15M
5M
10M
15M
Deoxypentitol
Glutamic acid
Heptadecanoic acid
Squalene
Beta-glutamic acid
3-Hydroxybutyric acid
Urea
Nicotinamide
2-Hydroxybutanoic acid
Alpha-aminoadipic acid
Glucoheptulose
Mannose
Fructose
Ribitol
Melezitose
Panose
Guanosine
Cis-gondoic acid
Palmitic acid
Valine
2-Ketoisocaproic acid
Oxalic acid
Tyrosine
N-acetylglutamate
Pseudouridine
1,5-Anhydroglucitol
Myo-inositol
Threonic acid
Succinic acid
3-Phosphoglycerate
Levoglucosan
Ethanolamine
Beta-alanine
Isoleucine
Leucine
Uracil
Succinate semialdehyde
Sorbitol
Galactitol
Xylitol
Pinitol
3,6-Anhydro-d-galactose
Glucose
Taurine
min
max

Fig. S21

Correlation analysis between the hepatic feature (76 transcripts and 44 metabolites that were commonly altered by diet, age and FXR KO) and serum/urine metabolites as well as cecal microbiota at genus level

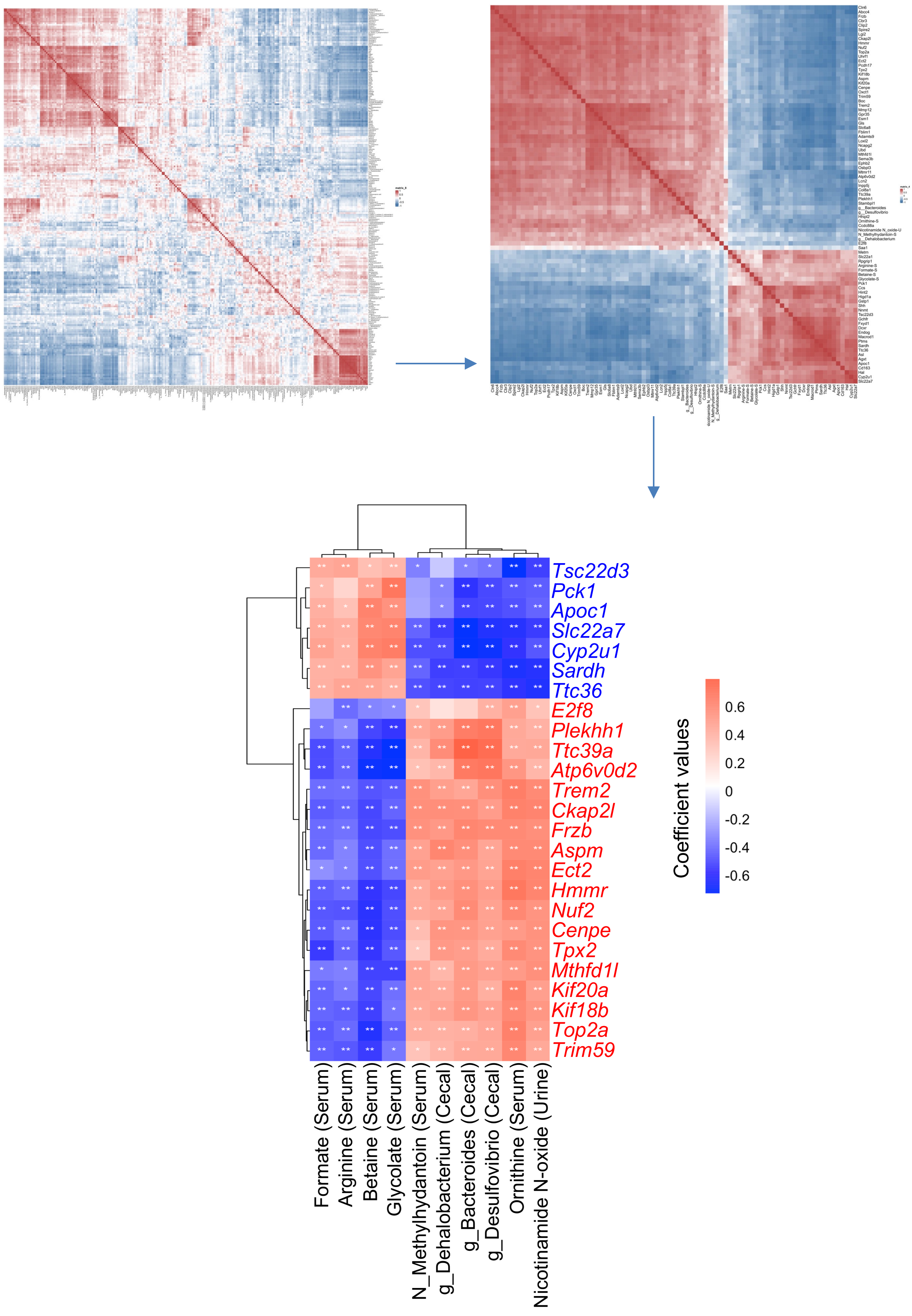

Fig. S22

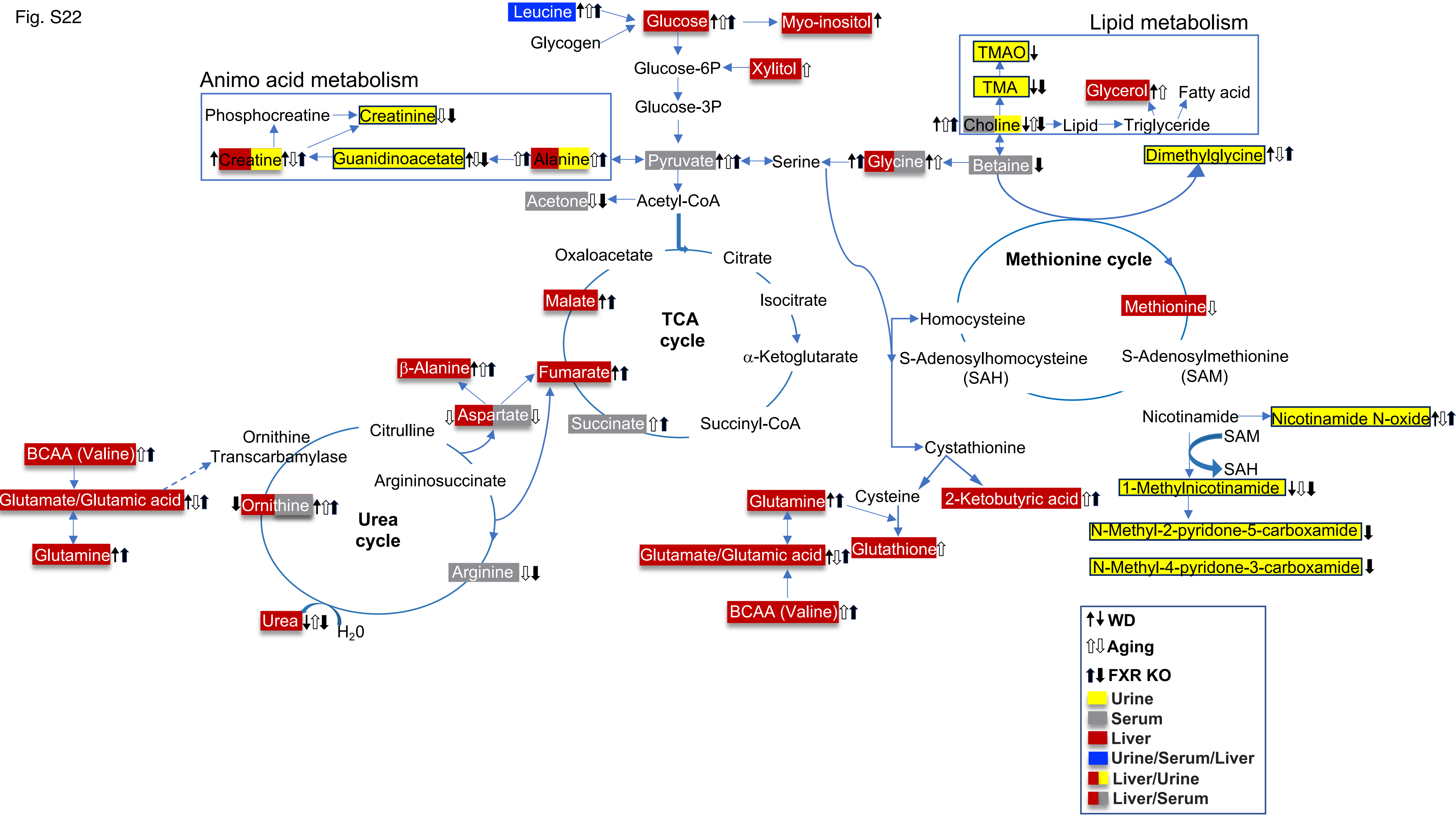

Leucine
Glucose
Glycogen
Myo-inositol
Glucose-6P
Xylitol
Glucose-3P
Lipid metabolism
TMAO
TMA
Glycerol
Fatty acid
Choline
Lipid
Triglyceride
Animo acid metabolism
Phosphocreatine
Creatinine
Creatine
Guanidinoacetate
Alanine
Pyruvate
Serine
Glycine
Betaine
Dimethylglycine
Acetone
Acetyl-CoA
Oxaloacetate
Citrate
Malate
TCA cycle
Isocitrate
α-Ketoglutarate
Methionine cycle
Methionine
Homocysteine
S-Adenosylhomocysteine (SAH)
S-Adenosylmethionine (SAM)
β-Alanine
Fumarate
Aspartate
Succinate
Succinyl-CoA
Nicotinamide
Nicotinamide N-oxide
SAM
SAH
Ornithine Transcarbamylase
Citrulline
BCAA (Valine)
Cystathionine
Argininosuccinate
Glutamate/Glutamic acid
Ornithine
Urea cycle
Glutamine
Cysteine
2-Ketobutyric acid
1-MethylInicotinamide
Glutathione
N-Methyl-2-pyridone-5-carboxamide
Glutamine
Arginine
Glutamate/Glutamic acid
N-Methyl-4-pyridone-3-carboxamide
BCAA (Valine)
Urea
H_2O
WD
Aging
FXR KO
Urine
Serum
Liver
Urine/Serum/Liver
Liver/Urine
Liver/Serum
