# Supplementary material for: The essential roles of FXR in diet and age influenced metabolic changes and liver disease development: a multi-omics study
Source: Biomark Res. 2023 Feb 18;11:20. doi: 10.1186/s40364-023-00458-9 (PMC9938992; doi:10.1186/s40364-023-00458-9)
Supplement: Supplementary file 3 — Additional file 3: Table S1. The function of hepatic transcripts that were consistently altered by WD intake in WT and FXR KO mice irrespective of their ages. [file 40364_2023_458_MOESM3_ESM.docx]

**Table S1. The function of transcripts that were consistently altered by WD in WT (36) and FXR KO (6) mice irrespective of their ages**

| Genes  (FXR dependent) | Description | WD *vs*. CD  (WT) | Function |
| --- | --- | --- | --- |
| *Adam11* | a disintegrin and metallopeptidase domain 11 | Up | Involved in cell-cell and cell-matrix interactions, including fertilization, muscle development, and neurogenesis |
| *Cyp3a11* | cytochrome P450, family 3, subfamily a, polypeptide 11 | Up | Oxidation-reduction process |
| *Gramd1b* | GRAM domain containing 1B | Up | Cholesterol transporter that mediates non-vesicular transport of cholesterol from the plasma membrane to the endoplasmic reticulum |
| *Cyp3a59* | cytochrome P450, family 3, subfamily a, polypeptide 59 | Up | Iron ion binding; oxidation-reduction process |
| *Orm3* | orosomucoid 3 | Up | Regulation of immune system process |
| *Pde5a* | Phosphodiesterase 5A | Up | Purine metabolism; Plays a role in signal transduction by regulating the intracellular concentration of cyclic nucleotides |
| *Slc39a4* | solute carrier family 39 (zinc transporter), member 4 | Up | Zinc influx into cells by the SLC39 gene family |
| *Adgrv1* | adhesion G protein-coupled receptor V1 | Up | Implicated in myoclonic epilepsy |
| *Sqle* | squalene epoxidase | Down | A key enzyme in cholesterol metabolism |
| *Glra3* | glycine receptor alpha 3 | Down | Ion transport |
| *Srgap3* | SLIT-ROBO Rho GTPase activating protein 3 | Down | Signal transduction; Positive regulation of GTPase activity |
| *D830032E09Rik* | RIKEN cDNA D830032E09 gene | Down | Unknown |
| *Mvd* | mevalonate diphosphate decarboxylase | Down | The enzyme mevalonate pyrophosphate decarboxylase catalyzes the conversion of mevalonate pyrophosphate into isopentenyl pyrophosphate in one of the early steps in cholesterol biosynthesis. It decarboxylates and dehydrates its substrate while hydrolyzing ATP. |
| *Ccdc69* | coiled-coil domain containing 69 | Down | Microtubule binding; Spindle midzone assembly |
| *Fdps* | Farnesyl diphosphate synthase | Down | It encodes an enzyme that catalyzes the production of geranyl pyrophosphate and farnesyl pyrophosphate from isopentenyl pyrophosphate and dimethylallyl pyrophosphate. |
| *Pdzk1ip1* | PDZK1 interacting protein 1 | Down | Plays an important role in tumor biology |
| *Nsdhl* | NAD(P) dependent steroid dehydrogenase-like | Down | A potential metastatic driver in triple-negative breast cancer |
| *Nat8f7* | N-acetyltransferase 8 (GCN5-related) family member 7 | Down | Pseudogene |
| *Acpp* | acid phosphatase, prostate | Down | It encodes an enzyme that catalyzes the conversion of orthophosphoric monoester to alcohol and orthophosphate. |
| *Rdh11* | retinol dehydrogenase 11 | Down | An NADPH-dependent retinal reductase and a short-chain dehydrogenase/reductase |
| *Gm32468* | predicted gene, 32468 | Down | Unknown |
| *Gm49012* | None | Down | Unknown |
| *Msmo1* | methylsterol monooxygenase 1 | Down | Cholesterol biosynthesis |
| *Lmntd2* | lamin tail domain containing 2 | Down | Involved in regulation of chromatin assembly |
| *Hamp2* | hepcidin antimicrobial peptide 2 | Down | Involved in the maintenance of iron homeostasis, and it is necessary for the regulation of iron storage in macrophages; antimicrobial activity against bacteria and fungi |
| *Tspyl4* | TSPY-like 4 | Down | Nucleosome assembly |
| *Cyp2c69* | cytochrome P450, family 2, subfamily c, polypeptide 69 | Down | Oxidation-reduction process |
| *Cyp2c40* | cytochrome P450, family 2, subfamily c, polypeptide 4 | Down | Oxidation-reduction process |
| *Fras1* | fraser extracellular matrix complex subunit 1 | Down | It encodes an extracellular matrix protein that appears to function in the regulation of epidermal-basement membrane adhesion and organogenesis during development. |
| *Fdft1* | farnesyl diphosphate farnesyl transferase 1 | Down | It encodes a membrane-associated enzyme located at a branch point in the mevalonate pathway. |
| *Tm7sf2* | transmembrane 7 superfamily member 2 | Down | Cholesterol biosynthetic process |
| *Gm10642* | predicted gene 10642 | Down | Unknown |
| *Fasn* | fatty acid synthase | Down | Catalyze the synthesis of palmitate from acetyl-CoA and malonyl-CoA, in the presence of NADPH, into long-chain saturated fatty acids. |
| *Nat8f6* | N-acetyltransferase 8 (GCN5-related) family member 6 | Down | Pseudogene |
| *Rec8* | REC8 meiotic recombination protein | Down | Meiotic synapsis |
| **Genes**  **(FXR-independent)** | **Description** | **WD *vs*. CD (FXR KO)** | **Function** |
| *Scd3* | stearoyl-coenzyme A desaturase 3 | Up | Rate-limiting enzyme catalyzing the synthesis of  monounsaturated fatty acids, mainly oleate (18:1) and palmitoleate (16:1) |
| *Cidec* | cell death-inducing DFFA-like effector c | Up | Encodes a member of the cell death-inducing DNA fragmentation factor-like effector family |
| *9130409I23Rik* | RIKEN cDNA 9130409I23 gene | Up | Lipid metabolic process; Sphingolipid biosynthetic process |
| *Csad* | cysteine sulfinic acid decarboxylase | Up | Organosulfur biosynthesis; Taurine biosynthesis |
| *Cyp39a1* | cytochrome P450, family 39, subfamily a, polypeptide 1 | Up | Steroid metabolism; Cholesterol degradation; Lipid metabolism; Bile acid biosynthesis |
| *Dntt* | deoxynucleotidyltransferase, terminal | Up | DNA metabolic process; DNA-dependent DNA replication |
