# Supplementary material for: The essential roles of FXR in diet and age influenced metabolic changes and liver disease development: a multi-omics study
Source: Biomark Res. 2023 Feb 18;11:20. doi: 10.1186/s40364-023-00458-9 (PMC9938992; doi:10.1186/s40364-023-00458-9)
Supplement: Supplementary file 4 — Additional file 4: Table S2. RNA sequence quality data. [file 40364_2023_458_MOESM4_ESM.docx]

**Table S2. RNA sequence quality data**

| Sample | Group (genotype-age-diet) | Raw reads | Effective (%) | Error (%) | Q20 (%) | Q30 (%) | GC (%) |
| --- | --- | --- | --- | --- | --- | --- | --- |
| A_5_1 | **WT-5-CD** | **47440490** | **98.6** | **0.02** | **98.49** | **95.66** | **48.47** |
| A_5_2 | WT-5-CD | 45897774 | 98.39 | 0.02 | 98.52 | 95.68 | 49.14 |
| A_5_3 | WT-5-CD | 55934524 | 98.58 | 0.02 | 98.44 | 95.32 | 48.87 |
| A_5_4 | WT-5-CD | 51748952 | 98.47 | 0.02 | 98.46 | 95.43 | 48.44 |
| A_10_19 | WT-10-CD | 68377230 | 98.12 | 0.02 | 98.66 | 96.14 | 49.35 |
| A_10_2 | WT-10-CD | 43538390 | 98.26 | 0.02 | 98.33 | 95.11 | 47.9 |
| A_10_3 | WT-10-CD | 45939292 | 98.9 | 0.02 | 98.39 | 95.24 | 47.88 |
| A_10_5 | WT-10-CD | 46155496 | 98.94 | 0.02 | 98.28 | 94.93 | 48.01 |
| A_15_1 | WT-15-CD | 49713604 | 98.53 | 0.02 | 98.64 | 96.02 | 48.79 |
| A_15_2 | WT-15-CD | 51609246 | 97.97 | 0.02 | 98.57 | 95.89 | 48.57 |
| A_15_3 | WT-15-CD | 45011236 | 98.25 | 0.02 | 98.57 | 95.84 | 48.85 |
| A_15_5 | WT-15-CD | 44083522 | 98.43 | 0.02 | 98.56 | 95.81 | 48.78 |
| B_5_1 | WT-5-WD | 48422084 | 98.45 | 0.02 | 98.46 | 95.34 | 48.5 |
| B_5_2 | WT-5-WD | 54483466 | 98.21 | 0.03 | 98 | 94.15 | 48.14 |
| B_5_3 | WT-5-WD | 45659764 | 98.2 | 0.02 | 98.54 | 95.68 | 47.55 |
| B_5_4 | WT-5-WD | 43857748 | 98.34 | 0.02 | 98.43 | 95.32 | 48.01 |
| B_10_B3 | WT-10-WD | 42019656 | 98.31 | 0.02 | 98.21 | 95 | 49.54 |
| B_10_B4 | WT-10-WD | 42508030 | 98.38 | 0.02 | 98.33 | 95.26 | 49.39 |
| B_10_B5 | WT-10-WD | 42578310 | 98.76 | 0.02 | 98.4 | 95.46 | 48.7 |
| B_10_B6 | WT-10-WD | 46265038 | 98.35 | 0.02 | 98.34 | 95.34 | 49.22 |
| B_15_1 | WT-15-WD | 41746894 | 98.39 | 0.02 | 98.54 | 95.81 | 49.16 |
| B_15_2 | WT-15-WD | 49613588 | 98.37 | 0.02 | 98.61 | 95.97 | 49.32 |
| B_15_3 | WT-15-WD | 41166428 | 98.32 | 0.02 | 98.62 | 96 | 49 |
| B_15_4 | WT-15-WD | 46705860 | 98.64 | 0.02 | 98.61 | 95.94 | 49.18 |
| C_5_1 | FXR KO-5-CD | 45363918 | 98.24 | 0.02 | 98.41 | 95.27 | 48.48 |
| C_5_2 | FXR KO-5-CD | 50452214 | 98.17 | 0.02 | 98.42 | 95.34 | 48.27 |
| C_5_3 | FXR KO-5-CD | 57903580 | 98.12 | 0.02 | 98.53 | 95.71 | 48.92 |
| C_5_4 | FXR KO-5-CD | 47437630 | 98.02 | 0.02 | 98.5 | 95.51 | 48.79 |
| C_10_C3 | FXR KO-10-CD | 40548056 | 98.98 | 0.02 | 98.18 | 94.96 | 49.42 |
| C_10_C4 | FXR KO-10-CD | 43376480 | 98.76 | 0.02 | 98.32 | 95.25 | 48.74 |
| C_10_C5 | FXR KO-10-CD | 44255628 | 98.59 | 0.02 | 98.44 | 95.55 | 49.05 |
| C_10_C6 | FXR KO-10-CD | 41625248 | 98.61 | 0.02 | 98.48 | 95.66 | 48.71 |
| C_15_1 | FXR KO-15-CD | 45102858 | 98.25 | 0.02 | 98.65 | 96.09 | 49.2 |
| C_15_2 | FXR KO-15-CD | 53791868 | 98.51 | 0.02 | 98.55 | 95.85 | 48.54 |
| C_15_3 | FXR KO-15-CD | 43671590 | 97.78 | 0.02 | 98.65 | 96.01 | 48.54 |
| C_15_4 | FXR KO-15-CD | 45878944 | 98.52 | 0.02 | 98.61 | 95.92 | 48.55 |
| D_5_1 | FXR KO-5-WD | 45014982 | 97.99 | 0.02 | 98.5 | 95.56 | 48.65 |
| D_5_2 | FXR KO-5-WD | 46641356 | 98.29 | 0.02 | 98.52 | 95.64 | 49.3 |
| D_5_3 | FXR KO-5-WD | 54108828 | 98.13 | 0.02 | 98.59 | 95.71 | 48.46 |
| D_5_4 | FXR KO-5-WD | 48965644 | 97.96 | 0.02 | 98.43 | 95.41 | 49.51 |
| D_10_2 | FXR KO-10-WD | 45884124 | 98.59 | 0.02 | 98.63 | 95.92 | 47.83 |
| D_10_7 | FXR KO-10-WD | 46826436 | 97.98 | 0.02 | 98.55 | 95.77 | 48.29 |
| D_10_D5 | FXR KO-10-WD | 43979422 | 98.44 | 0.02 | 98.44 | 95.55 | 49.63 |
| D_10_D6 | FXR KO-10-WD | 43187858 | 98.09 | 0.02 | 98.45 | 95.54 | 49.26 |
| D_15_1 | FXR KO-15-WD | 54403098 | 98.42 | 0.02 | 98.08 | 94.57 | 48.95 |
| D_15_2 | FXR KO-15-WD | 48213670 | 98.35 | 0.02 | 98.58 | 95.91 | 49.03 |
| D_15_3 | FXR KO-15-WD | 44217554 | 98.64 | 0.02 | 98.55 | 95.84 | 48.82 |
| D_15_4 | FXR KO-15-WD | 41950896 | 98.57 | 0.02 | 98.47 | 95.6 | 48.86 |

Effective: (Clean reads/Raw reads) * 100%

Error: base error rate

Q20, Q30: (Base count of Phred value > 20 or 30) / (Total base count)

GC: (G & C base count) / (Total base count)
